# Supplementary material for: TissUExM enables quantitative ultrastructural analysis in whole vertebrate embryos by expansion microscopy
Source: Cell Rep Methods. 2022 Sep 30;2(10):100311. doi: 10.1016/j.crmeth.2022.100311 (PMC9606133; doi:10.1016/j.crmeth.2022.100311)
Supplement: Document S1. Figures S1–S3 and Tables S1 and S2 [file mmc1.pdf]

Cell Reports Methods, Volume *2*

Supplemental information

TissUExM enables quantitative ultrastructural analysis in whole vertebrate embryos by expansion microscopy

Emmanuelle Steib, Rob Tetley, Romain F. Laine, Dominic P. Norris, Yanlan Mao, and Julien Vermot

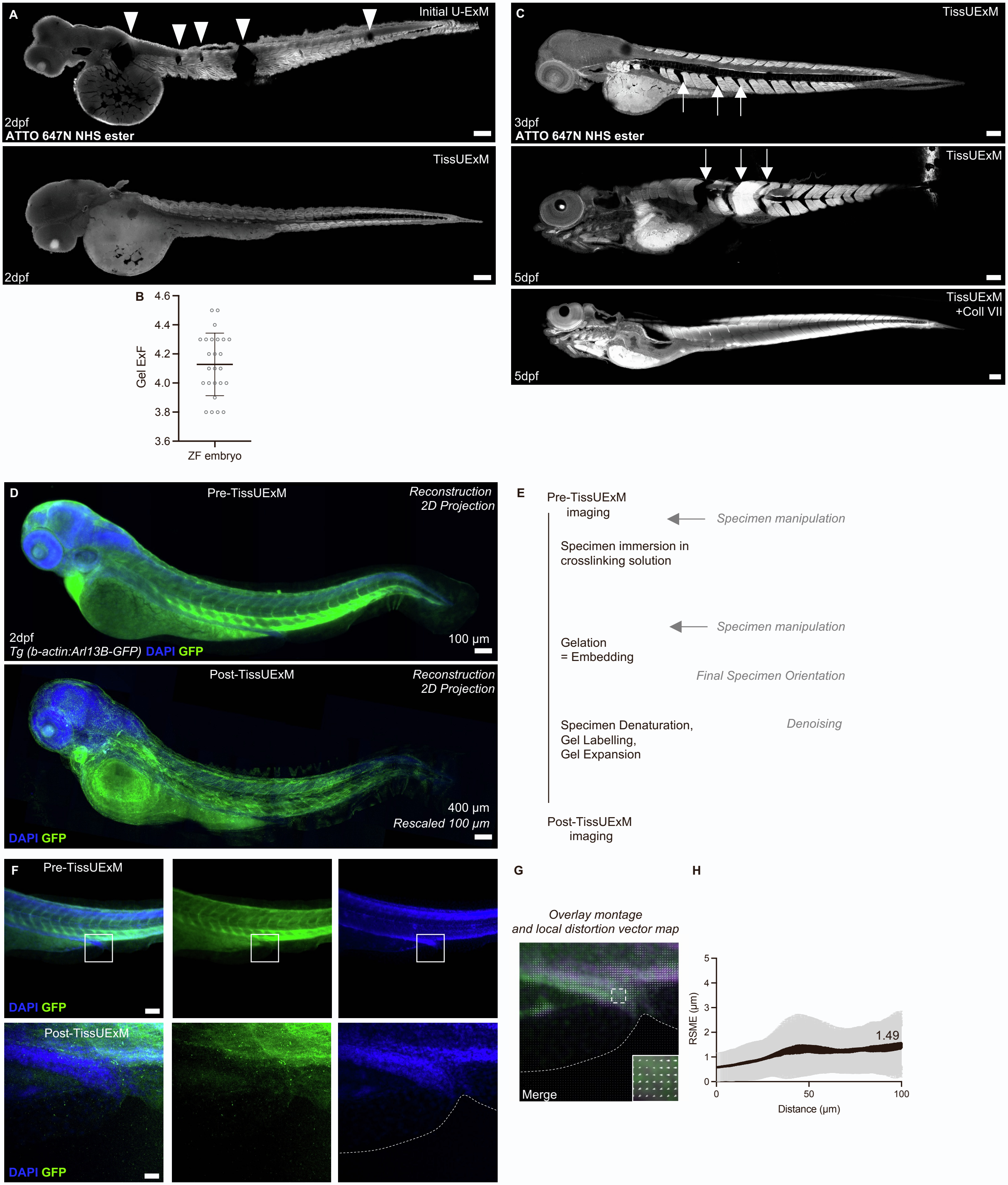

Supplemental Figure 1

Figure S1: TissUExM allows expansion of whole ZF embryos, Related to Figure 1

(A) 48hpf ZF processed with the initial U-ExM approach (top panel) or TissUExM (bottom panel), stained with ATTO 647N NHS-ester. White arrows point to cracking and specimen damage. Note that cracks are randomly localized. 10X/0.40 objective, scale bar: 100µm.
(B) Reproducibility of gel expansion factors for TissUExM processed 48hpf ZF embryo. Mean +/- SD: 4.1 +/-0.2, n=25 from 11 independent experiments.
(C) 3 to 5dpf ZF processed with basic TissUExM (top panels) or TissUExM+Collagenase VII digestion (bottom panel), stained with ATTO 647N NHS-ester. White arrows point to cracking and specimen damage. Note that cracks are restricted to myotendinous regions. 10X/0.40 objective, scale bar: 100µm, n=12 with 2 specimen/developmental stage/experiment, from 2 independent experiments.
(D) 48hpf Tg (b-actin:Arl13B-GFP) ZF imaged pre- (top panel) and post- (bottom panel) TissUExM, stained with DAPI. Entire specimen reconstruction and 2D-projection obtained from confocal imaging performed on individual tiles. 10X/0.40 objective. Pre-TissUExM corresponds to direct embryo imaging while post-TissUExM is obtained from gel-imaging. Note the appearance of the yolk-sac pre-TissUExM, with heterogenous protein density resulting from PFA-fixation.
(E) Pipeline recapitulating embryo manipulation during TissUExM.
(F) Example of the excretory canal as landmark pair for pre-(top) and post (bottom)-TissUExM distortion analysis. Inset in pre-TissUExM image corresponds to the expanded region displayed in the post-TissUExM image. 10X/0.40 objective, scale bars are respectively 100µm and 25µm.
(G) Overlay montage based on DAPI labelling, with post-TissUExM image automatically identified within pre-TissUExM image. Inset shows individual vectors for distortion analysis.
(H) Distortion analysis with individual Root Square Method Error (RSME, µm) for rescaled distance (µm). Mean+/- SD value 100µm away from centre: 1.49 +/- 0.9µm, n=20 pairs from two independent experiments. Note that RSME value at centre differs from 0 due to tilt in 3D specimen orientation between pre- and post-imaging.

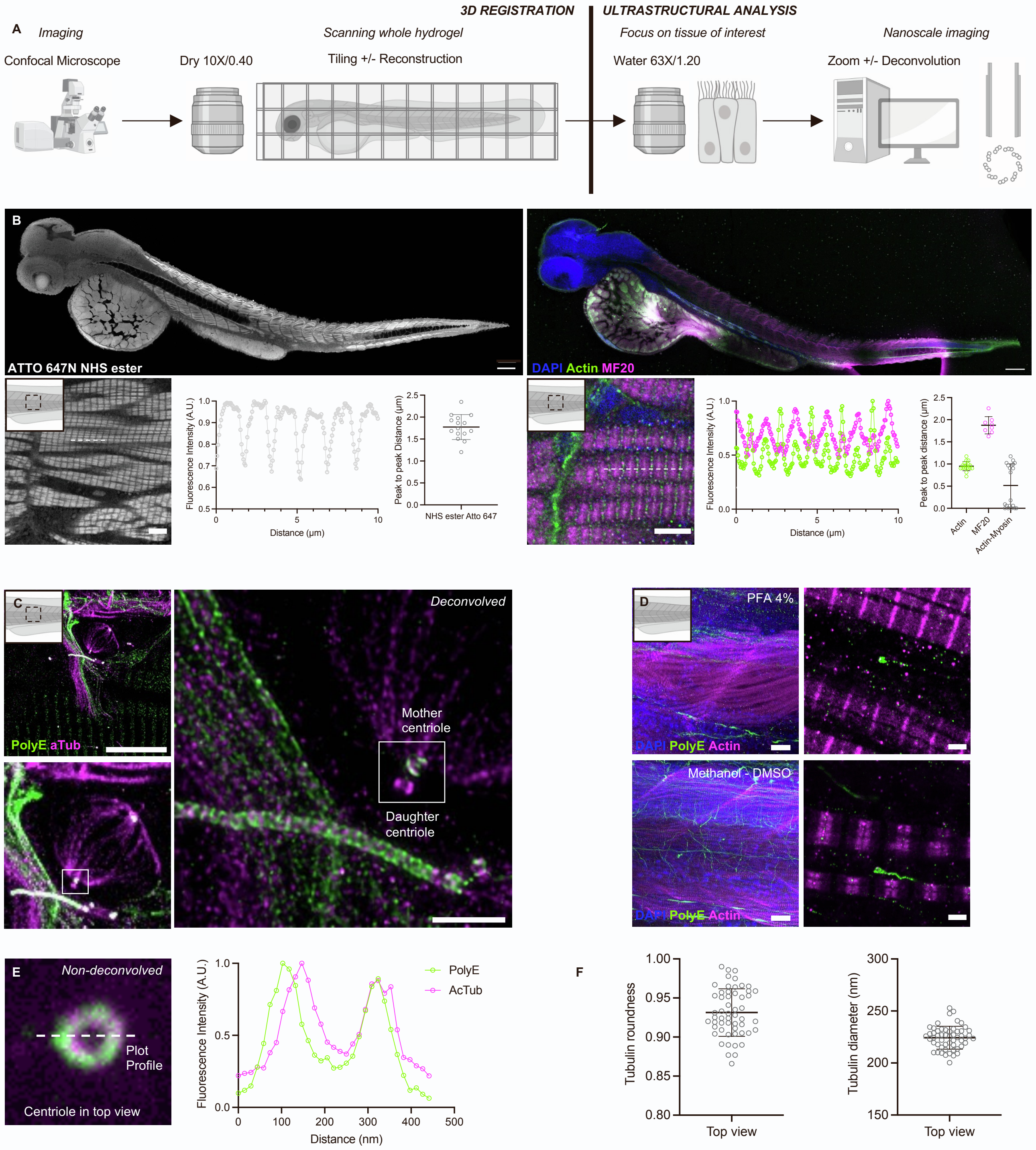

Supplemental Figure 2

Figure S2: TissUExM allows immunolabelling of whole ZF embryos and imaging at the nanoscale, Related to Figure 2

(A) Schematic representation of confocal imaging pipeline with respective objectives for different magnifications and degrees of details. Note that 63X working distance is limiting so multiple gel orientations should be considered. One gel can be imaged from different sides to maximize access to tissues of interest.
(B) TissUExM processed 48hpf ZF, labelled with ATTO 647N NHS ester (left panel) and immuno-stained for actin (green) and sarcomere myosin heavy chain (MF20, magenta) (right panel). 10X/0.40 objective, scale bar: 100µm. Inset on the sarcomeres and measurement of fluorescence intensity. Scale bar: 5µm. Mean +/- SD peak-to-peak distance with NHS labelling: 1.8 +/- 0.3 µm, n=15 distances from measures in three different sarcomeres. Mean +/- SD peak-to-peak distance with acto-myosin labelling: respectively between actin 0.9 +/-0.1µm, MF20 1.8 +/- 0.2µm and between acto-myosin.
(C) Trunk from TissUExM processed 48hpf ZF, stained for PolyE (green) and alpha-tubulin (magenta). Focus on mitotic cell near the spinal cord. Inset on deconvoluted image shows individual centrioles within the centrosome, with PolyE-positive mother centriole and PolyE-negative daughter centriole. 63X/1.20 objective, scale bars: 10µm, 1µm.
(D) Trunk from TissUExM-processed ZF embryos (10X), fixed at 48hpf either with PFA 4% (top panel) or Methanol 80%-DMSO 20% (bottom panel). Both specimen-embedded gels are processed in parallel and stained with PolyE (green) or Actin (magenta). Insets on sarcomeres and primary cilia (63X), showing that epitope conservation for individual antibodies is differentially affected by pre-TissUExM fixation, independently of gel processing. Scale bars: 20µm, 1µm.
(E) Representative TissUExM centriole in top view, with lumen resolved without deconvolution and associated plot profile.
(F) Validation of nanoscale isotropy. Mean +/- SD centriole roundness: 0.9313 +/- 0.03. n=54 centrioles from five independent experiments. Mean +/- SD centriole diameter: 224 +/- 11nm, n=54 centrioles from five independent experiments.

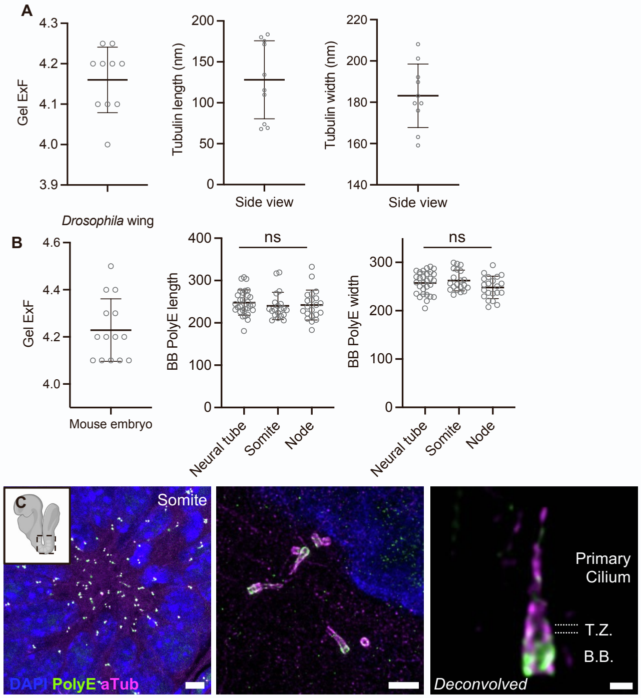

Supplemental Figure 3

Figure S3: TissUExM permits nanoscale expansion for different developmental models, Related to Figure 3

(A) Expansion validation in *Drosophila* wing discs. Reproducible expansion with Gel ExF mean +/- SD: 4.16+/- 0.1. n=10 gels from three independent experiments. Mean +/- SD centriolar tubulin length in side-view: 128+/- 48nm, n=10 centrioles from three independent experiments. Mean +/- SD centriolar tubulin width in side-view: 183 +/- 15nm, n=10 centrioles from three independent experiments.
(B) Expansion validation in mouse embryos. Reproducible expansion with Gel ExF mean +/- SD: 4.22 +/- 0.1. n=14 gels from six independent experiments. Mean +/- SD Basal body PolyE length respectively in neural tube 248 +/- 30nm, somite 240 +/- 32nm and node 242 +/- 35nm, n≥20 centriole/tissue from three independent experiments One-way ANOVA and Kruskal- Wallis ns p= 0.3532. Mean +/- SD Basal body PolyE width respectively in neural tube 257 +/- 23nm, somite 262 +/- 21nm and node 248 +/- 23nm, n≥20 centriole/tissue from three independent experiments One-way ANOVA ns p= 0.1235.
(C) Confocal imaging of a E8.5 mouse somite co-stained for PolyE (green) and a-tub (magenta). DAPI is in blue. From left to right: centre of the somite enriched in basal bodies (10X), multiple basal body/cilia complexes and inset on a primary cilium (63X). Scale bars respectively: 5µm, 1µm and 200nm.

Supplemental Tables

Protocol Name	Models	Whole organism	Isotropy mm-µm-nm	Commercial antibodies	High density labelling of endogenous proteins	Ultra-structure	Standard protocol across applications
Accepted publications							
ExM, Chen 2015	100µm mice brain sections, cultured cells	-	YES	-	-	YES	-
MAP, Ku 2016	Dissected organs from 8-weeks mice, cultured cells	-	Limited to mm-µm	YES	YES	-	-
ProExM, Tillberg 2016	100µm mice brain sections, 10µm human tissues sections, cultured cells,	-	YES	YES	-	YES	-
ExM zebrafish, Freifeld 2017	Dissected zebrafish heads	-	Limited to mm-µm	Limited	-	-	-
U-ExM, Gambarotto 2019	Isolated organelles, protists, cultured cells	-	Limited to µm-nm	YES	YES	YES	-
TRex, Damstra 2021	100µm mice brain sections, cultured cells	-	Limited to µm-nm	YES	YES	YES	-
Cryo-ExM, Laporte 2022	Cultured cells	-	Limited to µm-nm	YES	YES	YES	YES
Modified U-ExM, Mercey 2022	Mice retinal sections	-	Limited to µm-nm	YES	YES	YES	-
Preprints							
Whole ExM, Sim 2021, updated 2022	100µm mice brain sections, whole zebrafish embryos, whole mouse embryo	YES	Limited to mm-µm	Limited	-	-	-
TissUExM, This study	Whole zebrafish embryos, *Drosophila* wings, whole mouse embryo	YES	YES	YES	YES	YES	YES

Table S1: Summary description of major ExM protocols relevant for this study and rational for the development of TissUExM, Related to Figure 1

	Crosslinking	Embedding	Denaturation	Damage	Damage observation
U-ExM	1.4%FA, 2%AA - 5hrs	0.5% TEMED, 0.5% APS 5min + 1hr	90min - 95C	Major, irrespective of regions	Uncleared specimen, between gelation and denaturation, amplified by expansion
V2	↑AA concentration (20%)	↓ TEMED and APS ↑ Time	↓ Temperature ↑ Time (20hrs)	Important, mainly in the trunk	Uncleared specimen, between gelation and denaturation
V3	-	↑ 4-OH-TEMPO	-	Important, mainly in the trunk	Uncleared specimen, between gelation and denaturation
V4	↑Triton (0.1%)	↑Triton (0.1%)	-	Variable, restricted to deep trunk tissues	Cleared specimen, after expansion and NHS-labelling
V5	-	-	↑ Time (72hrs)	Variable, mostly in the centre of the tail	Cleared specimen, after expansion and NHS-labelling
V6	↑ Time (24hrs)	-	-	Minimal, restricted to the tail	Cleared specimen, after expansion and NHS-labelling
TissUExM	↑ Time (72hrs)	-	-	No damage	N/A

Table S2: Technical iterations to develop TissUExM , Related to STAR methods
