# Supplementary material for: TissUExM enables quantitative ultrastructural analysis in whole vertebrate embryos by expansion microscopy
Source: Cell Rep Methods. 2022 Sep 30;2(10):100311. doi: 10.1016/j.crmeth.2022.100311 (PMC9606133; doi:10.1016/j.crmeth.2022.100311)
Supplement: Document S2. Article plus supplemental information [file mmc4.pdf]

Cell Reports
Methods

Report

TissUExM enables quantitative ultrastructural analysis in whole vertebrate embryos by expansion microscopy

Graphical abstract

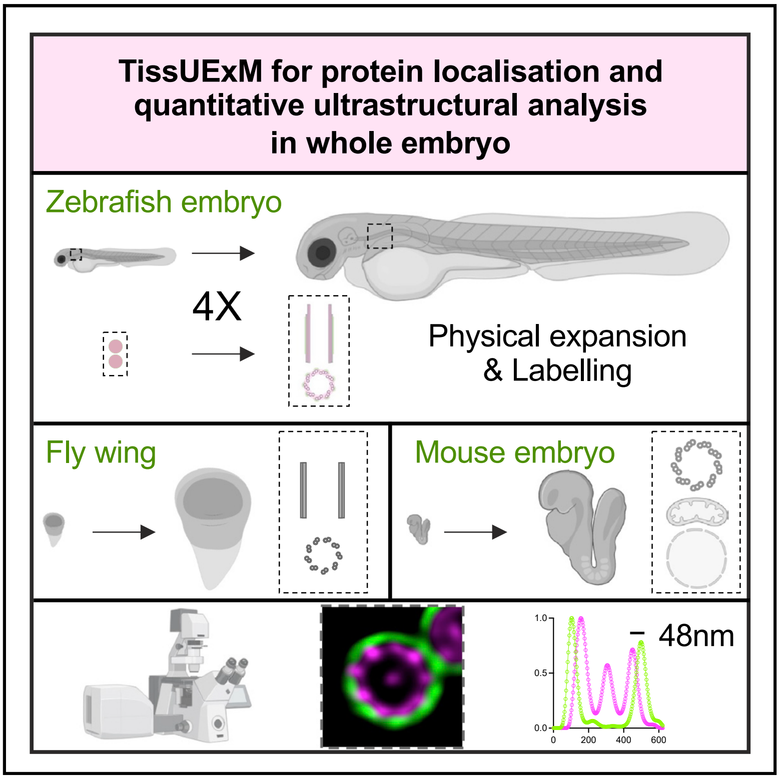

Authors

Emmanuelle Steib, Rob Tetley, Romain F. Laine, Dominic P. Norris, Yanlan Mao, Julien Vermot

Correspondence

j.vermot@imperial.ac.uk

In brief

Super-resolution microscopy is challenging in whole embryos, limiting structure-function analysis during developmental processes. Steib et al. developed TissUExM to physically expand heterogeneous tissues and label endogenous protein complexes. This approach can be applied to fly wings or to whole zebrafish and mouse embryos, enabling quantitative ultrastructural analysis at the nanometer scale.

Highlights

- We developed TissUExM for expansion microscopy of mechanically heterogeneous tissues
- TissUExM relies on post-expansion labeling for localization of endogenous proteins
- TissUExM facilitates quantitative ultrastructural analysis *in situ* in whole embryos
- TissUExM can be applied to whole zebrafish and mouse embryos or to fly wings

Steib et al., 2022, Cell Reports Methods 2, 100311
October 24, 2022 Crown Copyright © 2022
https://doi.org/10.1016/j.crmeth.2022.100311

CellPress

Cell Reports Methods

CellPress
OPEN ACCESS

Report

TissUExM enables quantitative ultrastructural analysis in whole vertebrate embryos by expansion microscopy

Emmanuelle Steib,[1] Rob Tetley,[2,4] Romain F. Laine,[2,5] Dominic P. Norris,[3] Yanlan Mao,[2] and Julien Vermot[1,6,*]
[1]Department of Bioengineering, Imperial College London, London SW7 2AZ, UK
[2]Laboratory for Molecular Cell Biology, University College London, London WC1E 6BT, UK
[3]MRC Harwell Institute, Mammalian Genetics Unit, Harwell Campus, Didcot OX11 0RD, UK
[4]Present address: Nikon UK Healthcare, 1 The Crescent, Surbiton KT6 4BN, UK
[5]Present address: Micrographia Bio, Translation & Innovation Hub, 84 Wood Lane, London W12 0BZ, UK
[6]Lead contact
*Correspondence: j.vermot@imperial.ac.uk
https://doi.org/10.1016/j.crmeth.2022.100311

MOTIVATION Super-resolution microscopy is challenging in whole embryos, which limits structure-function analysis during developmental processes. We developed a method allowing quantitative ultrastructural analysis in mechanically heterogeneous millimeter-scaled embryos, optimized for post-expansion labeling, and the localization of endogenous proteins in high-density environments.

SUMMARY

Super-resolution microscopy reveals the molecular organization of biological structures down to the nanoscale. While it allows the study of protein complexes in single cells, small organisms, or thin tissue sections, there is currently no versatile approach for ultrastructural analysis compatible with whole vertebrate embryos. Here, we present tissue ultrastructure expansion microscopy (TissUExM), a method to expand millimeter-scale and mechanically heterogeneous whole embryonic tissues, including *Drosophila* wing discs, whole zebrafish, and mouse embryos. TissUExM is designed for the observation of endogenous proteins. It permits quantitative characterization of protein complexes in various organelles at super-resolution in a range of ~3 mm-sized tissues using conventional microscopes. We demonstrate its strength by investigating tissue-specific ciliary architecture heterogeneity and ultrastructural defects observed upon ciliary protein overexpression. Overall, TissUExM is ideal for performing ultrastructural studies and molecular mapping *in situ* in whole embryos.

INTRODUCTION

Super-resolution (SR) microscopy has a profound impact on life sciences applications. Yet, SR is near impossible in tissues such as whole embryos due to large sample size and molecular crowding. Structure-function analysis is often limited to sections and remains challenging in mm-scale tissues. As a result, expansion microscopy (ExM) has emerged as a powerful alternative to discriminate fluorophores below the resolution limit of conventional microscopes (Chen et al., 2015; Chozinski et al., 2016; Tillberg and Chen, 2019; Truckenbrodt et al., 2019; Wassie et al., 2019).

In recent years, the approach has benefited from iterations tailored to individual models. There are currently two major expansion paths: ExM- and magnified analysis of the proteome (MAP)-derived protocols (Ku et al., 2016). ExM methods are based on specimen crosslinking with acroyl-X, pre-expansion labeling, and digestion with proteinase K (Damstra et al., 2022; Freifeld et al., 2017; Tillberg et al., 2016; Yu et al., 2020). MAP methods rely on acrylamide crosslinking, SDS and heat for specimen denaturation, and post-expansion labeling (Gambarotto et al., 2019; Laporte et al., 2022; Mao et al., 2020; M'Saad and Bewersdorf, 2020). For tissues, most methods were demonstrated on brain sections, which are relatively soft with low mechanical resistance to expansion.

Zebrafish (ZF) is a model of choice for human disease modeling and systems biology (Lieschke and Currie, 2007; Megason and Fraser, 2007; Pantazis and Supatto, 2014), which has proved challenging to expand. Only the ExM approach has been applied to date; however, it is suboptimal for staining efficiency and resolution of structures in crowded environments, like individual centrioles in centrosomes (Freifeld et al., 2017;

Crown Copyright © 2022
This is an open access article under the CC BY license (http://creativecommons.org/licenses/by/4.0/).

Sim et al., 2022). Moreover, protein mapping strongly relied on reporter lines for sufficient signal detection, limiting functional studies in wild-type and mutant strains.

It has been argued that MAP-derived approaches are best suited to localize endogenous protein complexes, especially in high molecular density environments. Three mechanisms underlie this: (1) post-expansion labeling benefits from better epitope access in crowded environments; (2) the linkage error from antibody size is downscaled based on expansion factor (Hamel and Guichard, 2021); and (3) the absence of proteinase K digestion, which is responsible for significant epitope loss (Yu et al., 2020). Ultrastructure ExM (U-ExM) is a MAP-derived method, calibrated for isotropic intracellular expansion, allowing accurate studies of physically resistant organelles such as centrioles. It also permits the mapping of molecular complexes with significantly higher resolution than with pre-expansion labeling (Gambarotto et al., 2019; Le Le Guennec et al., 2020; Hamel and Guichard, 2021; Steib et al., 2020). Yet, U-ExM is limited to thin sections, requiring critical adaptations for large and heterogeneous samples (Mercey et al., 2022).

We developed TissUExM, a versatile expansion method allowing quantitative imaging of endogenous molecules while preserving the larger three-dimensional (3D) environment. It enables accurate ultrastructural studies in heterogeneous tissues like entire vertebrate embryos (Figure 1A; Table S1).

RESULTS

Based on the established strengths of U-ExM in cells, we tested its performance in 2 days post-fertilization (dpf) ZF. While gel integrity seemed unaltered, obvious embryo cracking was observed, suggesting intra-specimen mechanical resistances (Figure S1A). As damage was heterogeneous, we reasoned that crosslinking, embedding, and denaturation were critical. We modified these steps (Table S2) and found that supplementation with 0.1% triton until embedding was essential for homogeneous penetration of chemicals. We also found that fixed embryos benefited from an increased acrylamide concentration at crosslinking. We optimized embedding by adapting the incubation time, the temperature, and the concentrations of polymerization initiators. Additionally, we decreased denaturation temperature to preserve most epitopes and increased time to inhibit intra-specimen resistance. This resulted in a successful expansion where we observed no damage in whole 2 dpf ZF (Figure S1A; Table S2), establishing the basis of TissUExM (expansion factor: 4.1 ± 0.2; Figure S1B).

As for most vertebrates, ZF from 3 dpf exhibit a resistant collagen network (Henry et al., 2005), incompatible with crosslinking and non-disruptive expansion. At 3 dpf, we observed characteristic damages restricted to myotendinous regions and not evident in the head, consistent with previous studies (Freifeld et al., 2017). As for *Drosophila* larvae and *C. elegans* cuticle (Jiang et al., 2018; Yu et al., 2020), we found that an additional collagenase VII digestion rescued protease-free 4-fold expansion of whole 5 dpf ZF. This step is easily added between gelation and denaturation for ZF at later developmental stages (Figure S1C).

We assessed TissUExM isotropy at the macroscale. We imaged embryos pre- and post-TissUExM and confirmed their morphology maintenance (Figure S1D). Yet, TissUExM of whole embryos requires specimen immersion and several manipulations (Figure S1E), making it difficult to retrieve the exact 3D orientation between pre- and post-TissUExM imaging. To quantify distortion at the μm-scale, we used landmark regions like the excretory canal or the tail (Figure S1F). Using an automated approach (Truckenbrodt et al., 2019), we observed less than 2.5% of distortion over 100 μm analyzed (root mean square error [RSME]: 1.49 ± 0.9 μm, Figures S1G and S1H), in line with the 1%–4% tolerated for whole organisms (Yu et al., 2020). We conclude that TissUExM preserves whole embryo morphology.

We combined ZF expansion with confocal imaging. With a 10×/0.40 numerical aperture (NA) objective, we assessed labeling homogeneity in the entire embryo and revealed biologically relevant information such as issue vascularization or innervation (Video S1). We next used a 63×/1.20 NA objective for ultrastructural analysis of regions of interest (Figure S2A). When working distance was limiting, the same gel was imaged from different sides, although specimen orientation must be considered at embedding. Alternatively, as for cleared tissues, we showed a satisfactory trade-off between tissue penetration and subcellular resolution using a 20×/0.75 NA dry objective (Video S2). To demonstrate SR in depth in large volumes, one would need a higher NA and longer working distance objective.

As control for labeling homogeneity, we used ATTO647N NHS-ester, a dye that binds to primary amines and permits visualization of bulk protein in a sample (Mao et al., 2020; M'Saad and Bewersdorf, 2020; Yu et al., 2020). We measured skeletal muscle sarcomere organization (NHS-Ester periodicity: 1.8 ± 0.3 μm) and recorded the expected sarcomere size (Squire, 2019). Although powerful for assessing tissue morphology, NHS-ester labeling is non-specific. We wished to specifically localize endogenous proteins, so we immunostained the same gel for actin and myosin heavy chain (myosin heavy chain antibody, MF20). In addition to the myosin periodicity (1.8 ± 0.2 μm), we measured the actin periodicity (0.9 ± 0.1 μm) that the NHS-ester labeling had not revealed (Figure S2B).

To further validate immunofluorescence, we co-stained a whole embryo for actin and polyglutamylated-tubulin (PolyE), a marker of stable microtubules enriched in neurons, as well as at centrioles and cilia (Janke and Magiera, 2020) (Figure 1B). This revealed the distributions of actin and PolyE in all tissues observed, demonstrating specific and homogeneous localization of endogenous cytoskeletal proteins, irrespectively of the region of interest. For example, the two centrioles in the centrosome of a mitotic cell from the trunk of the embryo were resolved (Figure S2C). Notably, this was achieved without a fluorescent line. We conclude that TissUExM resolves endogenous structures in high molecular density environments.

As with conventional immunofluorescence, ExM relies on sample fixation. We evaluated the impact of fixation and epitope loss on TissUExM by comparing two classic ZF fixatives, 4% PFA and Dent's (methanol 80%, DMSO 20%) (Figure S2D). We excluded glutaraldehyde fixation as it distorts morphology in deep tissues (Copper et al., 2018). We co-stained for actin and PolyE, and while both fixations resulted in similar expansion factors and specimen integrity, we observed antibody-specific differences in fluorescence intensity and homogeneity. Focusing

CellPress
OPEN ACCESS

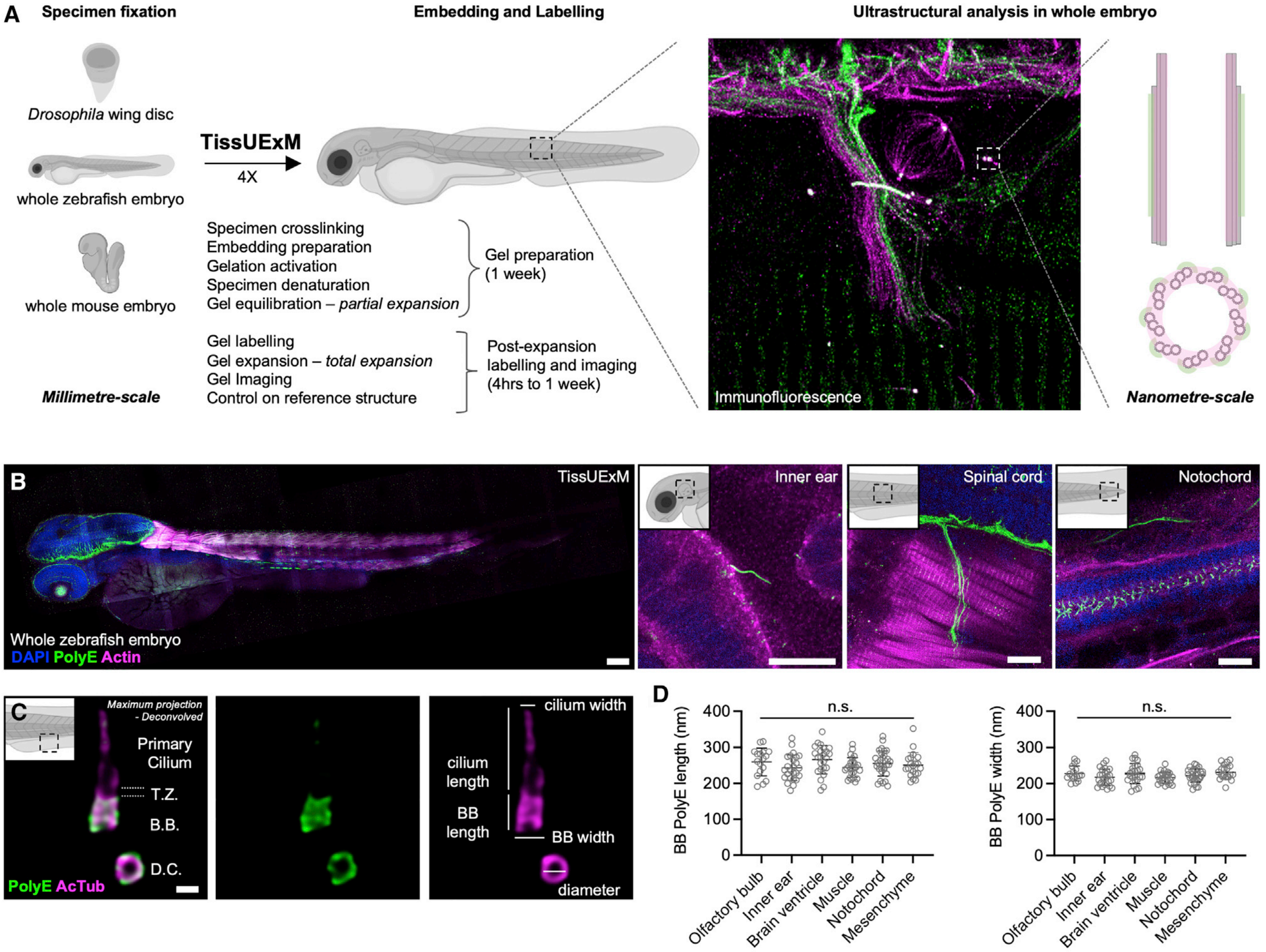

Figure 1. TissUExM allows quantitative super-resolution imaging of endogenous proteins *in situ*

(A) Schematic summary of TissUExM.

(B) Two dpf ZF stained for PolyE and Actin. DAPI is in blue. Left to right: whole embryo, inner ear and kinocilium on tether cell, spinal cord and sarcomeres, and notochord with cilia. 10×/0.40, scale bars (gel ExF rescaled): 100, 10, 10, and 10 μm.

(C) BB-primary cilium complex in side view, with daughter centriole in top view, stained for PolyE and AcTub. T.Z. stands for transition zone, B.B. for basal body, and D.C. for daughter centriole. Note that PolyE is restricted to the central core of the BB. 63×/1.20, scale bar: 200 nm.

(D) BB PolyE in ciliated cells from various tissues. Mean ± SD length in olfactory bulb: 259 ± 38 nm; inner ear: 243 ± 36 nm; brain ventricle: 266 ± 27 nm; muscle: 255 ± 35 nm; notochord: 255 ± 35 nm; and mesenchyme: 251 ± 37 nm. n $\geq$ 17 BBs/tissue from six independent experiments. Normality by Kolmogorov-Smirnov, one-way ANOVA ns p = 0.1960. Mean ± SD width in olfactory bulb: 228 ± 21 nm; inner ear: 218 ± 22 nm; brain ventricle: 228 ± 28 nm; muscle: 216 ± 15 nm; notochord: 222 ± 19 nm; and mesenchyme: 231 ± 20. n $\geq$ 17 BBs/tissue from six independent experiments. Normality by Kolmogorov-Smirnov, one-way ANOVA ns p = 0.1104.

See also Figures S1 and S2.

on ciliated muscle cells, we detected stronger actin network staining in PFA-fixed ZF, while microtubule-based cilia were more homogenously labeled after Dent's fixation. As a result of gains in resolution from TissUExM, artifact detection may be enhanced compared with conventional microscopy. Moreover, targeting a protein with a monoclonal or polyclonal antibody can give rise to slight differences at the nanoscale (Figures S2A and S2D). Together, these results suggest that TissUExM performs equally well on PFA- or methanol-fixed ZF, allowing researchers to use the fixative of choice for their organelle or antibody of expertise.

ExM protocols must be carefully controlled to validate isotropic intracellular expansion, which is crucial for ultrastructural studies. To test nanoscale accuracy, we imaged basal bodies (BBs; also known as mature centrioles) in various organs throughout the expanded embryo and measured their dimensions and roundness (Figure 1C). We used BBs as molecular rulers, as they display an evolutionarily conserved 9-fold microtubule-triplet symmetry, forming a ~450 nm-long and ~220 nm-wide barrel. Staining for PolyE, we visualized the BB core (Hamel et al., 2017) and measured its length and width in six representative tissues: olfactory bulb, inner ear, brain ventricle, muscle,

notochord, and mesenchyme. To visualize the entire BB-cilium complex, we co-stained with acetylated-tubulin (AcTub) or alpha-tubulin (aTub). We found that BB architecture was conserved in each of the six tissues (PolyE length: 255 ± 35 nm; Figure 1D), corresponding to U-ExM results in protists and human cells (Gambarotto et al., 2019; Le Guennec et al., 2020). Since the cylindrical nature of centrioles was visible, we measured their characteristic roundness and diameter when imaged in perfect top view (roundness: 0.93 ± 0.03, diameter: 224 ± 11 nm; Figures S2E and S2F), demonstrating that TissUExM preserves macromolecular complexes architecture *in situ*. Overall, we confirm that TissUExM generates linear expansion of the entire embryo, regardless of the tissue considered, thereby facilitating ultrastructural studies at the nanometer scale *in situ*.

As an example of application, we focused on cilia, which are important organelles involved in human disease, regulating fluid motion and signaling pathways. Patients with ciliopathy exhibit tissue-specific defects that remain poorly understood and require studies in whole developing organisms (Reiter and Leroux, 2017). Due to their small diameter (~200 nm), structural analyses of ciliary defects have relied on electron microscopy (Papon et al., 2010), thereby uncoupling them from mechanistic studies based on specific protein localization.

We used TissUExM to study ciliary heterogeneity in whole embryos (Figure 2A). We analyzed the hair cells from the lateral line and the olfactory bulb, which carry motile cilia. Coupling TissUExM with deconvolution, we observed ultrastructural details previously limited to EM in ZF, like the 9-fold symmetry and the axoneme's central pair (Figures 2B and 2C). We next analyzed the muscles and mesenchyme for their primary cilia. Surprisingly, we found that primary cilia did not display the textbook 9 + 0 microtubule-doublet architecture, characterized by homogeneous tubulin width along the cilium. Rather, we observed a rapid tubulin thinning along the proximodistal axis, marked either with PolyE or AcTub (Figure 2D). We quantified significant diameter differences between motile and primary cilia (Figure 2E), confirming cryo-electron tomography observations made *in cellulo* (Kiesel et al., 2020; Sun et al., 2019).

To rule out an artifact of post-translational tubulin modification, we stained for aTub (Figure 2F). We analyzed the inner ear and the notochord, which display a mix of motile and primary cilia and where ciliogenesis and ciliary disassembly are very dynamic (Colantonio et al., 2009). While PolyE was ubiquitous in motile cilia, we observed clear variability in primary cilia labeling, with tissue-specific heterogeneity (Figures 2G and 2H). Overall, TissUExM revealed a significant heterogeneity in axonemal architecture directly in control embryos.

Next, we investigated the effects of ciliary protein overexpression. We analyzed a GFP-reporter line ubiquitously overexpressing ADP-ribosylation factor-like protein 13B (Arl13B) in embryonic tissues (Borovina et al., 2010). Arl13B localizes to the ciliary membrane and is required for ciliogenesis, cilia maintenance, and ciliary signaling (Fisher et al., 2020). We detected GFP at the ciliary membrane and validated that TissUExM is compatible with GFP-reporter lines (Figure 2I). Strikingly, TissUExM revealed that Arl13b-GFP overexpression leads to ciliary malformations that include axonemal bifurcation and abnormal accumulation of ciliary membrane (Figure 2J). Together, TissUExM allowed quantitative characterization of ciliary ultrastructure in whole embryos and is well suited to revealing subtle architectural disorganization of the microtubule shaft and the ciliary envelope.

We further assessed TissUExM amenability to study other species. We used *Drosophila* larval wing imaginal discs, which express myosin II regulatory light chain-GFP (Royou et al., 2004), and demonstrated efficient expansion and labeling of the cytoskeleton (gel ExF: 4.2 ± 0.1) (Figures 3A and S3A). As in ZF, we focused on centrosomes, known to display short non-canonical centrioles in fly (Greenan et al., 2018). We confirmed TissUExM nanoscale accuracy, observing round centrioles (roundness 0.93 ± 0.4) of 175 ± 9 nm in diameter, in agreement with previous SR observations on isolated fly cells (Fu and Glover, 2012).

Similarly, we analyzed whole-mount embryonic day 8.5 (E8.5) mouse embryos, expanded with reproducible 4-fold expansion (gel ExF: 4.2 ± 0.1; Figure S3B). We observed centrioles with the expected dimensions, roundness, and diameter in various embryonic regions (Figures 3C, 3D, S3B, and S3C), confirming that TissUExM can be used on other whole vertebrate embryos. Importantly, we detected similar ultrastructural differences to those in ZF between motile node cilia and primary cilia from the neural tube and somites (Shinohara and Hamada, 2017). Mouse primary cilia displayed a proximodistal axonemal thinning, suggesting a general structural feature of primary cilia axonemes in embryonic vertebrate tissues (Figure S3C).

Finally, we evaluated TissUExM to study the subcellular morphology of a range of organelles at SR in tissues. We stained mouse embryos and successfully imaged the outer mitochondrial membranes (Translocase of Outer Mitochondrial Membrane [TOMM20]), the vesicular and membrane accumulation of clathrin heavy chain (clathrin HC), and the Golgi apparatus (Golgi Matrix protein 130 kD [GM130]) and resolved individual nuclear pores (nucleoporin 205 kD [NUP205]). However, we did not resolve the nuclear pore lumen, as NUP205 is part of the pore inner ring and below TissUExM resolution limit (Kosinski et al., 2016). Notably, we targeted endogenous epitopes, which differs from previous studies using overexpressed and tagged nucleoporins in cells, generating a brighter and larger fluorescent ring (Thevathasan et al., 2019). Further, we successfully imaged tight junctions (zonula occludens-1 [ZO-1]) and the nuclear envelope protein Lamin B1 (Figure 3E). Each organelle was imaged at resolutions below 200 nm in whole embryos, without fluorescent tags or deconvolution. We conclude that TissUExM is a versatile method for researchers to study organelles in multiple types of metazoan tissues.

DISCUSSION

We developed TissUExM as a versatile ExM method to preserve ultrastructural details in heterogeneous tissues while allowing the labeling of endogenous proteins in high molecular density environments. It is applicable to multiple major developmental biology models, i.e., fly, ZF, and mouse. Using BBs as reference structures, we demonstrated linear isotropic intracellular expansion. We also showed TissUExM performance on various

CellPress
OPEN ACCESS

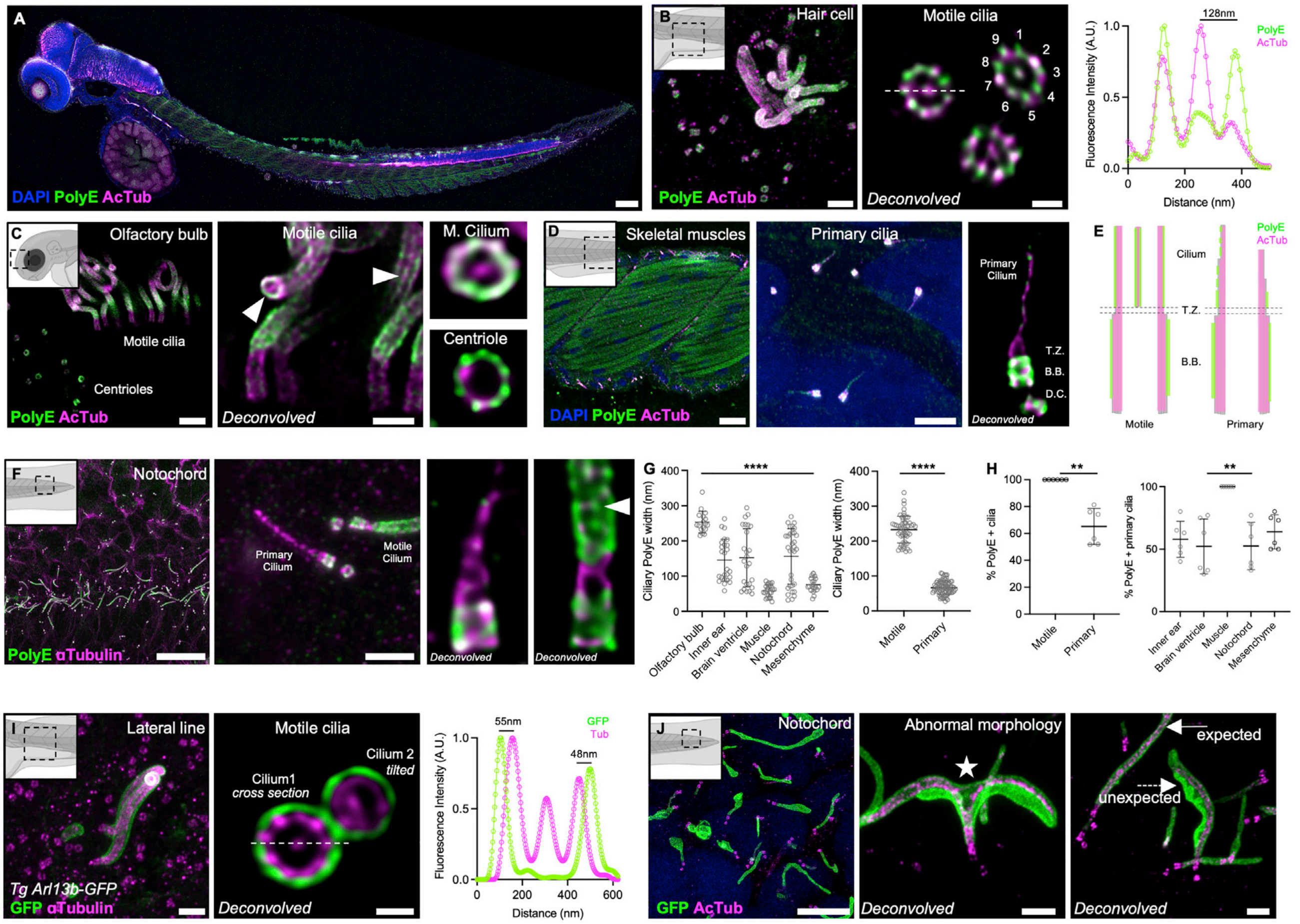

Figure 2. TissUExM reveals tissue specific diversity of ciliary architecture

(A) Two dpf ZF stained for PolyE and AcTub. DAPI is in blue. 10×/0.40, scale bar: 100 μm.

(B) Hair cell with centrioles and motile cilia, fluorescence intensity profile across the cilium (63X). Scale bars: 1 μm and 200 nm.

(C) Olfactory bulb with motile cilia in side view or motile cilium and centriole in top views (63X). White arrow points to the central pair. Scale bars: 1 μm and 500nm.

(D) Skeletal muscles (10×) and mesenchyme with insets on primary cilia (63×). Scale bars: 20 μm and 1 μm.

(E) Schematic differences between motile and primary cilia axonemes. Pink coverage for tubulin acetylation, and green for polyglutamylation.

(F) Notochord of 2 dpf ZF, stained for PolyE and aTub (10×). Inset on region with a PolyE-primary cilium and a PolyE + motile cilium (63×). White arrow points to the central pair. Scale bars: 10 and 1 μm.

(G) Tissue-specific ciliary sizes. Mean ± SD ciliary PolyE length in olfactory bulb: 4,095 ± 1,060 nm; inner ear: 1,055 ± 1,498 nm; brain ventricle: 1,612 ± 1,715 nm; muscle: 1,210 ± 406 nm; notochord: 1,798 ± 1,112 nm; and mesenchyme: 1,147 ± 446 nm. n ≥ 17 cilia/tissue from six independent experiments. One-way ANOVA and Kruskal-Wallis ****$p < 0.0001$. Mean ± SD ciliary PolyE width in olfactory bulb: 253 ± 31 nm; inner ear: 145 ± 60 nm; brain ventricle: 152 ± 83 nm; muscle: 58 ± 17 nm; notochord: 156 ± 79 nm; and mesenchyme: 76 ± 21 nm. n ≥ 17 cilia/tissue from six independent experiments. One-way ANOVA and Kruskal-Wallis ****$p < 0.0001$. Mean ± SD ciliary PolyE width in motile cilia: 233 ± 39 nm versus primary cilia: 66 ± 19 nm. n = 54 motile cilia and n = 78 primary cilia, from six independent experiments. Normality validated by Kolmogorov-Smirnov, Welch t test ****$p < 0.0001$.

(H) Tissue-specific ciliary polyglutamylation. Mean ± SD percentage of PolyE + motile cilia: 100% ± 0% versus primary cilia: 65% ± 13%. n = 54 motile cilia and n = 78 primary cilia, from six independent experiments. Mann-Whitney **$p = 0.0022$. Mean ± SD percentage of PolyE + primary cilia in inner ear 58% ± 14%; brain ventricle: 52% ± 22%; muscle 100% ± 0%; notochord: 52% ± 19%; and mesenchyme: 64% ± 14%. n = 60 cilia/tissue from six independent experiments. One-way ANOVA and Kruskal-Wallis **$p = 0.0042$. Mean ± SD percentage of PolyE + primary cilia in various tissues, either co-stained with AcTub, inner ear: 69% ± 9%; brain ventricle: 72% ± 6%; notochord: 69% ± 8%; and mesenchyme 76% ± 4%, or co-stained with aTub, inner ear: 46% ± 6%; brain ventricle: 33% ± 2%; notochord: 36% ± 3%; and mesenchyme: 52% ± 3%. n = 30 cilia/tissue from three independent experiments. One-way ANOVA with multiple comparison ****$p < 0.0001$. Kruskal-Wallis ns $p = 0.7777$ on AcTub primary cilia and **$p = 0.0030$ on aTub primary cilia **$p = 0.0030$.

(I and J) Two dpf Arl13B-GFP ZF.

(I) Hair cell stained for GFP and aTub. Inset on perfect top view across a motile cilium, with fluorescence intensity profile. 63×/1.20, scale bars: 1 μm and 200 nm.

(J) Notochord (10×), stained for GFP and AcTub. Insets on abnormal cilia (63×) with white star for axonemal bifurcation and white arrows showing ultrastructural differences with ciliary membrane accumulation. Scale bars: 5, 1, and 1 μm.

See also Figure S2.

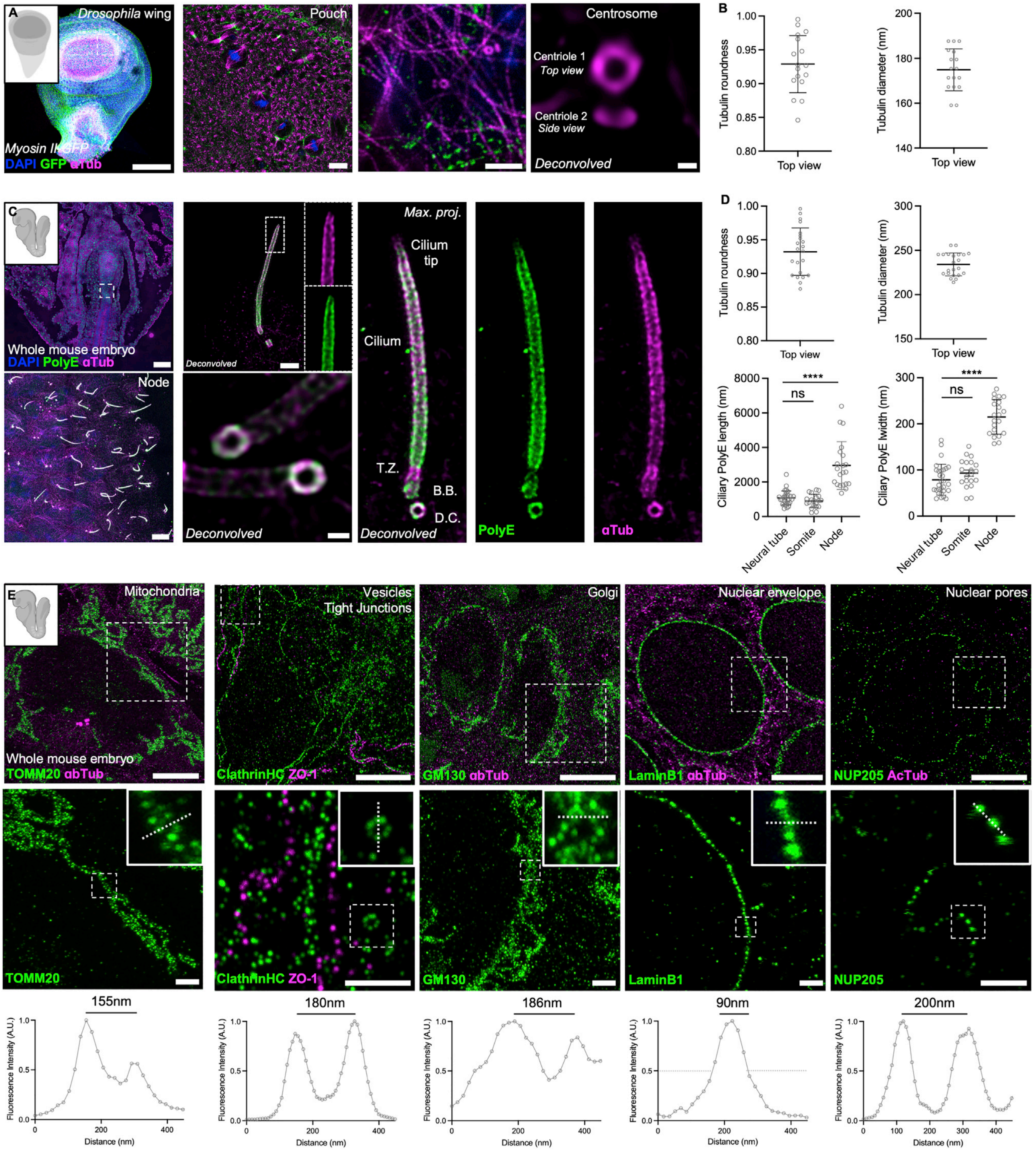

Figure 3. TissUExM is a versatile method for various models and organelles

(A) 110 h post-fertilization (hpf) myosin II regulatory light chain-GFP *Drosophila* wing disc, stained for GFP and a-tub; DAPI is in blue. Whole wing (10×), apical side of the pouch with dividing cells (10×), microtubule cytoskeleton (63×), and deconvolved centrosome with a pair of centrioles in top and side views (63×). Scale bars: 100, 5, 1, and 100 nm.

(B) Centrioles in top view. Mean ± SD tubulin roundness: 0.929 ± 0.042. n = 17 centrioles from three independent experiments. Mean ± SD tubulin diameter: 175 ± 9 nm. n = 17 centrioles from three independent experiments.

(legend continued on next page)

organelles, providing their maintenance after chemical fixation. Recently, U-ExM of cultured cells confirmed well-described chemical fixations artifacts, in contrast to cryo-fixation (Laporte et al., 2022). To date, high-pressure freezing remains limited to ~500 μm-large tissues and has not yet been successfully applied to whole vertebrate embryos. It will be interesting to reassess our method once this milestone is reached.

TissUExM allowed us to clarify ciliary diversity in various organs, as well as pathological ciliary features. It appears particularly well suited for the study of primary cilia *in vivo* and opens new avenues for this field. Overall, TissUExM should prove invaluable in systems biology as well as for the study of congenital diseases in whole animal and other types of heterogeneous samples including organoids and biopsies.

Limitations of the study

We validated TissUExM on centrioles and cilia, which are stable beyond most fixations. We do not rule out that other ExM methods give better results for specific organelles or antibodies of interest. Researchers may need to compare protocols for their field of expertise.

STAR★METHODS

Detailed methods are provided in the online version of this paper and include the following:

- KEY RESOURCES TABLE
- RESOURCE AVAILABILITY
  - ○ Lead contact
  - ○ Materials availability
  - ○ Data and code availability
- EXPERIMENTAL MODEL AND SUBJECT DETAILS
  - ○ Animal studies
- METHOD DETAILS
  - ○ Initial U-ExM gel preparation and optimization on ZF
  - ○ TissUExM of ZF and mouse embryos
  - ○ Collagenase VII digestion
  - ○ TissUExM of *D. melanogaster* wings
- QUANTIFICATION AND STATISTICAL ANALYSIS
  - ○ Isotropy validation at the macroscale in ZF embryo
  - ○ Distortion analysis
  - ○ Isotropy validation at the nanoscale in ZF and mouse embryos
  - ○ Measurements of ciliary features in ZF and mouse
  - ○ Measurement in *D. melanogaster* wings
  - ○ Measurements of fluorescence intensity for staining in mouse embryos
  - ○ Statistics and reproducibility

SUPPLEMENTAL INFORMATION

Supplemental information can be found online at https://doi.org/10.1016/j.crmeth.2022.100311.

ACKNOWLEDGMENTS

We thank the Vermot Lab, especially Christina Vagena-Pantoula, for help with ZF husbandry. We thank Jonathan Whitchurch for providing fixed mouse embryos. E.S. is funded by the Marie Sklodovska Curie Action Program (H2020-MSCA-IF-2020, GA101028893). R.T. was funded by MRC award MR/T031646/1, and Y.M. was funded by Lister Institute Research Prize. Y.M. was also funded by MRC awards MR/L009056/1 and MR/T031646/1, and EMBO Young Investigator Programme. This work was also supported by MRC funding to the MRC LMCB University Unit at UCL, award code MC_U12266B. D.P.N. is funded by UK Medical Research Council (MC_U142670370). J.V. is funded by the European Research Council (ERC) under the European Union's Horizon 2020 research and innovation program (GA682938).

AUTHOR CONTRIBUTIONS

E.S. and J.V. conceived and designed the project. E.S. designed, performed, and analyzed all the experiments of the manuscript, with additional help from R.T. for *Drosophila* wing preparation and imaging. R.F.L. performed the distortion analysis. Y.M. and D.P.N. provided expertise to perform image analysis. J.V. supervised the project. E.S. and J.V. wrote the manuscript, with the help of all the co-authors.

DECLARATION OF INTERESTS

The authors declare no competing interests.

Received: January 13, 2022
Revised: June 11, 2022
Accepted: September 12, 2022
Published: September 30, 2022

REFERENCES

Borovina, A., Superina, S., Voskas, D., and Ciruna, B. (2010). Vangl2 directs the posterior tilting and asymmetric localization of motile primary cilia. Nat. Cell Biol. *12*, 407–412. https://doi.org/10.1038/ncb2042.

Chen, F., Tillberg, P.W., and Boyden, E.S. (2015). Expansion microscopy. Science *347*, 543–548. https://doi.org/10.1126/science.1260088.

Chozinski, T.J., Halpern, A.R., Okawa, H., Kim, H.-J., Tremel, G.J., Wong, R.O.L., and Vaughan, J.C. (2016). Expansion microscopy with conventional

(C) E8.5 whole mouse, stained for PolyE and aTub. DAPI is in blue. Top left: whole embryo (10×); bottom left: node enriched in cilia (10×); top right: maximum projection of a node cilium with inset on the tip (63×); bottom right: maximum projection and deconvolution applied to two cilia with magnification of the cilium base (63×). Right panel shows a BB/cilium complex architecture with subregions such as the TZ and the cilium tip. Scale bars: 100, 1, and 5μm and 200 nm.
(D) Centrioles and cilia in top view. Mean ± SD tubulin roundness: 0.932 ± 0.035. n = 22 centrioles from three independent experiments. Mean ± SD tubulin diameter: 234 ± 13 nm. n = 22 centrioles from three independent experiments. Mean ± SD ciliary PolyE length in neural tube: 1,075 ± 411 nm, somite: 899 ± 385 nm, and node 2,956 ± 1,384 nm. n ≥ 20 cilia/tissue from three independent experiments. One-way ANOVA and Kruskal-Wallis ****$p < 0.0001$. Mean ± SD ciliary PolyE width in neural tube: 79 ± 33 nm, somite: 93 ± 30 nm, and node: 215 ± 37 nm. n ≥ 20 cilia/tissue from three independent experiments. One-way ANOVA and Kruskal-Wallis ****$p < 0.0001$.
(E) Organelles in E8.5 mouse. Mitochondria (TOMM20), vesicles (clathrin heavy chain) and tight junctions (ZO1), Golgi apparatus (GM130), nuclear envelope (Lamin B1), and nuclear pore complexes (NUP205). 63×/1.20, Scale bars: 5 and 1 μm. Insets show the regions for fluorescence intensity profiles. Note that no deconvolution was applied.
See also Figure S3.

antibodies and fluorescent proteins. Nat. Methods *13*, 485–488. https://doi.org/10.1038/nmeth.3833.

Colantonio, J.R., Vermot, J., Wu, D., Langenbacher, A.D., Fraser, S., Chen, J.-N., and Hill, K.L. (2009). The dynein regulatory complex is required for ciliary motility and otolith biogenesis in the inner ear. Nature *457*, 205–209. https://doi.org/10.1038/nature07520.

Copper, J.E., Budgeon, L.R., Foutz, C.A., van Rossum, D.B., Vanselow, D.J., Hubley, M.J., Clark, D.P., Mandrell, D.T., and Cheng, K.C. (2018). Comparative analysis of fixation and embedding techniques for optimized histological preparation of zebrafish. Comp. Biochem. Physiol., Part C: Toxicol. Pharmacol. *208*, 38–46, 8th Aquatic Models of Human Disease Conference held at the University of Alabama at Birmingham, in January, 2017. https://doi.org/10.1016/j.cbpc.2017.11.003.

Damstra, H.G.J., Mohar, B., Eddison, M., Akhmanova, A., Kapitein, L.C., and Tillberg, P.W. (2022). Visualizing cellular and tissue ultrastructure using tenfold robust expansion microscopy (TREx). Elife *11*, e73775. https://doi.org/10.7554/eLife.73775.

Fisher, S., Kuna, D., Caspary, T., Kahn, R.A., and Sztul, E. (2020). ARF family GTPases with links to cilia. Am. J. Physiol. Cell Physiol. *319*, C404–C418. https://doi.org/10.1152/ajpcell.00188.2020.

Freifeld, L., Odstrcil, I., Förster, D., Ramirez, A., Gagnon, J.A., Randlett, O., Costa, E.K., Asano, S., Celiker, O.T., Gao, R., et al. (2017). Expansion microscopy of zebrafish for neuroscience and developmental biology studies. Proc. Natl. Acad. Sci. USA *114*, E10799–E10808. https://doi.org/10.1073/pnas.1706281114.

Fu, J., and Glover, D.M. (2012). Structured illumination of the interface between centriole and peri-centriolar material. Open Biol. *2*, 120104. https://doi.org/10.1098/rsob.120104.

Gambarotto, D., Zwettler, F.U., Le Guennec, M., Schmidt-Cernohorska, M., Fortun, D., Borgers, S., Heine, J., Schloetel, J.-G., Reuss, M., Unser, M., et al. (2019). Imaging cellular ultrastructures using expansion microscopy (U-ExM). Nat. Methods *16*, 71–74. https://doi.org/10.1038/s41592-018-0238-1.

Greenan, G.A., Keszthelyi, B., Vale, R.D., and Agard, D.A. (2018). Insights into centriole geometry revealed by cryotomography of doublet and triplet centrioles. Elife *7*, e36851. https://doi.org/10.7554/eLife.36851.

Hamel, V., and Guichard, P. (2021). Chapter 14 - improving the resolution of fluorescence nanoscopy using post-expansion labeling microscopy. In Methods in Cell Biology, Expansion Microscopy for Cell Biology, P. Guichard and V. Hamel, eds. (Academic Press), pp. 297–315. https://doi.org/10.1016/bs.mcb.2020.07.002.

Hamel, V., Steib, E., Hamelin, R., Armand, F., Borgers, S., Flückiger, I., Busso, C., Olieric, N., Sorzano, C.O.S., Steinmetz, M.O., et al. (2017). Identification of Chlamydomonas central core centriolar proteins reveals a role for human WDR90 in ciliogenesis. Curr. Biol. *27*, 2486–2498.e6. https://doi.org/10.1016/j.cub.2017.07.011.

Henry, C.A., McNulty, I.M., Durst, W.A., Munchel, S.E., and Amacher, S.L. (2005). Interactions between muscle fibers and segment boundaries in zebrafish. Dev. Biol. *287*, 346–360. https://doi.org/10.1016/j.ydbio.2005.08.049.

Janke, C., and Magiera, M.M. (2020). The tubulin code and its role in controlling microtubule properties and functions. Nat. Rev. Mol. Cell Biol. *21*, 307–326. https://doi.org/10.1038/s41580-020-0214-3.

Jiang, N., Kim, H.J., Chozinski, T.J., Azpurua, J.E., Eaton, B.A., Vaughan, J.C., and Parrish, J.Z. (2018). Superresolution imaging of Drosophila tissues using expansion microscopy. Molecular Biology of the Cell *29*, 1413–1421. https://doi.org/10.1091/mbc.E17-10-0583.

Kiesel, P., Alvarez Viar, G., Tsoy, N., Maraspini, R., Gorilak, P., Varga, V., Honigmann, A., and Pigino, G. (2020). The molecular structure of mammalian primary cilia revealed by cryo-electron tomography. Nat. Struct. Mol. Biol. *27*, 1115–1124. https://doi.org/10.1038/s41594-020-0507-4.

Kosinski, J., Mosalaganti, S., von Appen, A., Teimer, R., DiGuilio, A.L., Wan, W., Bui, K.H., Hagen, W.J.H., Briggs, J.A.G., Glavy, J.S., et al. (2016). Molecular architecture of the inner ring scaffold of the human nuclear pore complex. Science *352*, 363–365. https://doi.org/10.1126/science.aaf0643.

Ku, T., Swaney, J., Park, J.-Y., Albanese, A., Murray, E., Cho, J.H., Park, Y.-G., Mangena, V., Chen, J., and Chung, K. (2016). Multiplexed and scalable super-resolution imaging of three-dimensional protein localization in size-adjustable tissues. Nat. Biotechnol. *34*, 973–981. https://doi.org/10.1038/nbt.3641.

Laporte, M.H., Klena, N., Hamel, V., and Guichard, P. (2022). Visualizing the native cellular organization by coupling cryofixation with expansion microscopy (Cryo-ExM). Nat. Methods *19*, 216–222. https://doi.org/10.1038/s41592-021-01356-4.

Lieschke, G.J., and Currie, P.D. (2007). Animal models of human disease: zebrafish swim into view. Nat. Rev. Genet. *8*, 353–367. https://doi.org/10.1038/nrg2091.

Le Guennec, M., Klena, N., Gambarotto, D., Laporte, M.H., Tassin, A.-M., van den Hoek, H., Erdmann, P.S., Schaffer, M., Kovacik, L., Borgers, S., et al. (2020). A helical inner scaffold provides a structural basis for centriole cohesion. Sci. Adv. *6*, eaaz4137. https://doi.org/10.1126/sciadv.aaz4137.

Mao, C., Lee, M.Y., Jhan, J.-R., Halpern, A.R., Woodworth, M.A., Glaser, A.K., Chozinski, T.J., Shin, L., Pippin, J.W., Shankland, S.J., et al. (2020). Feature-rich covalent stains for super-resolution and cleared tissue fluorescence microscopy. Sci. Adv. *6*, eaba4542. https://doi.org/10.1126/sciadv.aba4542.

Megason, S.G., and Fraser, S.E. (2007). Imaging in systems biology. Cell *130*, 784–795. https://doi.org/10.1016/j.cell.2007.08.031.

Mercey, O., Kostic, C., Bertiaux, E., Giroud, A., Sadian, Y., Gaboriau, D.C.A., Morrison, C.G., Chang, N., Arsenijevic, Y., Guichard, P., and Hamel, V. (2022). The connecting cilium inner scaffold provides a structural foundation that protects against retinal degeneration. PLoS Biol. *20*, e3001649. https://doi.org/10.1371/journal.pbio.3001649.

M'Saad, O., and Bewersdorf, J. (2020). Light microscopy of proteins in their ultrastructural context. Nat. Commun. *11*, 3850. https://doi.org/10.1038/s41467-020-17523-8.

Pantazis, P., and Supatto, W. (2014). Advances in whole-embryo imaging: a quantitative transition is underway. Nat. Rev. Mol. Cell Biol. *15*, 327–339. https://doi.org/10.1038/nrm3786.

Papon, J.F., Coste, A., Roudot-Thoraval, F., Boucherat, M., Roger, G., Tamalet, A., Vojtek, A.M., Amselem, S., and Escudier, E. (2010). A 20-year experience of electron microscopy in the diagnosis of primary ciliary dyskinesia. Eur. Respir. J. *35*, 1057–1063. https://doi.org/10.1183/09031936.00046209.

Reiter, J.F., and Leroux, M.R. (2017). Genes and molecular pathways underpinning ciliopathies. Nat. Rev. Mol. Cell Biol. *18*, 533–547. https://doi.org/10.1038/nrm.2017.60.

Royou, A., Field, C., Sisson, J.C., Sullivan, W., and Karess, R. (2004). Reassessing the role and dynamics of nonmuscle myosin II during furrow formation in early Drosophila embryos. Mol. Biol. Cell *15*, 838–850. https://doi.org/10.1091/mbc.e03-06-0440.

Schindelin, J., Arganda-Carreras, I., Frise, E., Kaynig, V., Longair, M., Pietzsch, T., Preibisch, S., Rueden, C., Saalfeld, S., Schmid, B., et al. (2012). Fiji: an open-source platform for biological-image analysis. Nat. Methods *9*, 676–682. https://doi.org/10.1038/nmeth.2019.

Sim, J., Park, C.E., Cho, I., Min, K., Eom, M., Han, S., Jeon, S., Cho, H.-J., Cho, E.-S., Kumar, A., et al. (2022). Nanoscale resolution imaging of the whole mouse embryos and larval zebrafish using expansion microscopy. Preprint at bioRxiv. https://doi.org/10.1101/2021.05.18.443629.

Shinohara, K., and Hamada, H. (2017). Cilia in left–right symmetry breaking. Cold Spring Harb. Perspect. Biol. *9*, a028282. https://doi.org/10.1101/cshperspect.a028282.

Squire, J. (2019). Special issue: the actin-myosin interaction in muscle: background and overview. Int. J. Mol. Sci. *20*, 5715. https://doi.org/10.3390/ijms20225715.

Steib, E., Laporte, M.H., Gambarotto, D., Olieric, N., Zheng, C., Borgers, S., Olieric, V., Le Guennec, M., Koll, F., Tassin, A.-M., et al. (2020). WDR90 is a centriolar microtubule wall protein important for centriole architecture integrity. Elife *9*, e57205. https://doi.org/10.7554/eLife.57205.

Sun, S., Fisher, R.L., Bowser, S.S., Pentecost, B.T., and Sui, H. (2019). Three-dimensional architecture of epithelial primary cilia. Proc. Natl. Acad. Sci. USA *116*, 9370–9379. https://doi.org/10.1073/pnas.1821064116.

Thevathasan, J.V., Kahnwald, M., Cieśliński, K., Hoess, P., Peneti, S.K., Reitberger, M., Heid, D., Kasuba, K.C., Hoerner, S.J., Li, Y., et al. (2019). Nuclear pores as versatile reference standards for quantitative superresolution microscopy. Nat. Methods *16*, 1045–1053. https://doi.org/10.1038/s41592-019-0574-9.

Tillberg, P.W., and Chen, F. (2019). Expansion microscopy: scalable and convenient super-resolution microscopy. Annu. Rev. Cell Dev. Biol. *35*, 683–701. https://doi.org/10.1146/annurev-cellbio-100818-125320.

Tillberg, P.W., Chen, F., Piatkevich, K.D., Zhao, Y., Yu, C.C.J., English, B.P., Gao, L., Martorell, A., Suk, H.J., Yoshida, F., et al. (2016). Protein-retention expansion microscopy of cells and tissues labeled using standard fluorescent proteins and antibodies. Nat. Biotechnol. *34*, 987–992. https://doi.org/10.1038/nbt.3625.

Truckenbrodt, S., Sommer, C., Rizzoli, S.O., and Danzl, J.G. (2019). A practical guide to optimization in X10 expansion microscopy. Nat. Protoc. *14*, 832–863. https://doi.org/10.1038/s41596-018-0117-3.

Wassie, A.T., Zhao, Y., and Boyden, E.S. (2019). Expansion microscopy: principles and uses in biological research. Nat. Methods *16*, 33–41. https://doi.org/10.1038/s41592-018-0219-4.

Yu, C.C.J., Barry, N.C., Wassie, A.T., Sinha, A., Bhattacharya, A., Asano, S., Zhang, C., Chen, F., Hobert, O., Goodman, M.B., et al. (2020). Expansion microscopy of C. elegans. Elife *9*, e46249. https://doi.org/10.7554/eLife.46249.

STAR★METHODS

KEY RESOURCES TABLE

REAGENT or RESOURCE	SOURCE	IDENTIFIER
Antibodies		
PolyE, Rabbit poly.	Adipogen	Cat#AG-25B-0030-C050, pab (IN105)
Actin, Mouse mono.	IGBMC	Cat#ACT-2D7, alpha actin
Actin, Rabbit poly.	Sigma	Cat#A2066
Acetylated tubulin	Thermofisher	Cat#32-2700; RRID:AB_2533073
Alpha-tubulin, Mouse mono.	Life technologies	Cat#62204; RRID:AB_1965960
Beta-tubulin, Mouse mono.	Life technologies	Cat#322600; RRID:AB_86547
Myosin heavy chain, Mouse mono	DHSB	Cat#MF20
GFP, Rabbit poly.	Thermofisher	Cat#A11122; RRID:AB_221569
TOMM20, Rabbit poly.	Life technologies	Cat#PA552843; RRID:AB_2648808
GOLGA2/GM130, Rabbit poly.	Proteintech	Cat#11308-1-AP, Ag1848; RRID:AB_2115327
Clathrin Heavy Chain, Rabbit poly.	Abcam	Cat#Ab21679; RRID:AB_2083165
NUP205, Rabbit poly.	Proteintech	Cat#24439-1-AP, Ag19832; RRID:AB_2879550
Lamin B1, Rabbit mono.	Abcam	Cat#Ab16048; RRID:AB_443298
anti-rabbit Alexa 488, Goat	Thermofisher	Cat#A-11008; RRID:AB_143165
anti-mouse Alexa 568, Goat	Thermofisher	Cat#A-11004; RRID:AB_2534072
Chemicals, peptides, and recombinant proteins		
1-phenyl-2-thiourea (PTU)	Sigma	Cat#P7629
Paraformaldehyde (PFA, 16%)	Fisher Scientific	Cat#28908
Methanol	Sigma	Cat#34860
DMSO	Sigma	Cat#D2650
Triton X100	Fisher Scientific	Cat#10102913
Formaldehyde (FA, 36.5–38%)	Sigma	Cat#F8775
Acrylamide (AA, 40%)	Sigma	Cat#A4058
N,N′-methylbisacrylamide (bis-AA, 2%)	Sigma	Cat#M1533
Sodium acrylate (SA, 97–99%)	Sigma	Cat#408220
4-hydroxy-TEMPO	Sigma	Cat#176141
Ammonium persulfate (APS)	Thermofisher	Cat#17874
Tetramethylethylenediamine (TEMED)	Thermofisher	Cat#17919
Sodium dodecyl sulfate (SDS)	Sigma	Cat#L3771
Tris	Merck	Cat#T1503
Tween20	Merck	Cat#P1379
Phosphate Buffer Saline	Merck	Cat#D8537
Poly-D-Lysine (PDL, 1 mg/mL)	Gibco	Cat#A3890401
12mm coverslip, Menzel	Thermofisher	Cat#11846933
24mm coverslip, Menzel	Thermofisher	Cat#11817892
Attofluor chamber	Thermofisher	Cat#A7816
ATTO 647N-NHS	Sigma	Cat#18373
Experimental models: Organisms/strains		
Zebrafish: WT/AB	Zebrafish International Resource Center (ZIRC)	ZFIN: ZDB-GENO-960809-7

(*Continued on next page*)

Continued

REAGENT or RESOURCE	SOURCE	IDENTIFIER
Zebrafish: Tg(b-actin:Arl13B-GFP)	Borovina et al., 2010	ZDB-ALT-100721-1
Drosophila melanogaster	Royou et al., 2004	sqh-GFP
Mouse	Mary Lyon Centre, UK	C57BL/6N
Software and algorithms		
Fiji	Schindelin et al., 2012	https://imagej.github.io/
Prism v9Graph	GraphPad	https://www.graphpad.com

RESOURCE AVAILABILITY

Lead contact

Further information and requests for resources and reagents should be directed to and will be fulfilled by the lead contact, Julien Vermot (j.vermot@imperial.ac.uk).

Materials availability

This study did not generate new unique reagents.

Data and code availability

- All data reported in this paper will be shared by the lead contact upon request.
- This paper does not report original code.
- Any additional information required to reanalyse the data reported in this paper is available from the lead contact upon request.

EXPERIMENTAL MODEL AND SUBJECT DETAILS

Animal studies

Zebrafish husbandry, use of transgenic lines and fixation

All experiments using ZF were performed following the European directive 2010/63/EU and Home Office guidelines under the project licence was PP6020928. WT/AB or *Tg(b-actin:Arl13b-GFP)* fish were in-crossed to generate embryos clutches, raised at 28.5°C in 1X Danieau's buffer and treated with 0.003% 1-phenyl-2-thiourea (PTU) at 20hrs to prevent pigment formation.

By default, ZF embryos were fixed in PFA 4% in PBS at RT for 6hrs with orbital agitation. Embryos were then gradually dehydrated in a methanol series (25%, 50%, 75%, 100%) for 10 min at RT, then stored at −20°C. Alternatively, ZF embryos were fixed in Dent's fixative (−20°C; Methanol 80%-DMSO 20%) then stored in pure methanol at −20°C.

D. melanogaster *husbandry, wings dissection and fixation*

Fly stocks were raised on standard cornmeal molasses fly food medium at 25°C. Per 1L, the fly food contained 10g agar, 15g sucrose, 33g glucose, 35 g years, 15g maize meal, 10g wheat germ, 30g treacle, 7.22g soya flour, 1g nipagin, 5mL propionic acid.

To visualise non-muscle Myosin II, we used flies of the genotype sqhAX3; sqh-GFP (Royou et al., 2004). Third instar (approximately 110hr AEL) larval wing imaginal discs were dissected out from larvae using forceps in Shields and Sang M3 media (Merck) supplemented with 2% FBS (Merck), 1% pen/strep (Gibco), 3 ng/ml ecdysone (Merck) and 2 ng/ml insulin (Merck). Wing discs were fixed for 10 minutes in 18% formaldehyde (Merck) diluted in PBS prior to being washed 4 × 10 minutes in PBT (PBS, 0.3% Triton X-100) and rinsed 4 times in PBS. All steps were carried out with gentle rocking. Wing discs were stored in PBS at 4°C prior to expansion.

Mouse husbandry, surgery, and fixation

Mouse embryos were collected under guidance from the MRC Harwell Ethics Committee and the UK Home Office; Euthanasia was by cervical dislocation. The sex of embryos was not determined - outside of a slight male-specific growth advantage, morphological sex differences are not evident at the development stage analysed. C57BL/6N intercrosses were set up at the Mary Lyon Centre, MRC Harwell Institute.

Pregnancy was assessed by vaginal plugs. Pregnant C57BL/6N females were culled by cervical dislocation and 8.5 days post coitum (dpc) embryos dissected in PBS under a light microscope. Embryos were fixed for 12–16 hours in 4%PFA in PBS, dehydrated in a methanol series (30%, 50%, 75%, 100%), then stored at −20°C.

Mice were housed in groups of 2–5 with controlled temperature (21 ± 2 C) and humidity (55 ± 10%) in a 12-hour light/dark cycle. Mice had free access to water and were fed *ad libitum* on a commercial diet (Special Diet Services, UK).

METHOD DETAILS

Initial U-ExM gel preparation and optimization on ZF

U-ExM was first applied to 2dpf ZF similarly to Le Guennec et al. (2020). In brief, 4% PFA fixed embryos were incubated for 5hrs in 2% AA; 1.4%FA at 37°C, then washed once in PBS and set for gelation in 'initial' activated monomer solution (19% SA; 10% AA; 0.1% bis-AA; 0.5% TEMED; 0.5% APS) for 5min on ice then 1 hr at 37°C in a humid chamber. Gels were transferred to 35mm dishes filled with denaturation buffer for 15min at RT, then 1.5mL tubes for 90min at 95°C. At the end of denaturation, gels were washed 2X in PBS then expanded 3 × 30min in ddH20. Expanded gels were immersed in an excess of ATTO 647N NHS-Ester/H20 (1:1000) for 1 hr at RT, then washed 3 × 30min with ddH20. Morphological integrity of the gel-embedded embryos was assessed post-gelation and post-expansion. Each step of the protocol was adapted until no obvious morphological damage was observed (Table S2).

TissUExM of ZF and mouse embryos

Fixed embryos were first rehydrated in gradual concentrations of 25%–50%-75% PBS/Methanol at RT prior to incubation of individual embryos in 2mL-tubes in 500µL of crosslinking solution (20% AA; 1.4% FA; 0.1% triton) for 72 hrs at 37°C.

Each embryo was washed 3 × 5min in an excess of PBS-Triton 0.1% and incubated in 90µL inactivated monomer solution (21% SA; 11% AA; 0.1% bis-AA; 0.1% triton) overnight at 4°C. Embryos were washed 3 × 5min in an excess of PBS-Triton 0.1% and placed on parafilm in a humid chamber for gelation in 50µL of activated monomer solution (19% SA; 10% AA; 0.1% bis-AA; 0.1% triton; 0.01% 4-OH-TEMPO; 0.25% TEMED; 0.25% APS) covered by a 12mm-coverslip, then incubated 1 hr at 4°C followed by 2 hrs at 37°C. Note that the differences in final concentrations between inactivated and activated monomer solution results from diluting 90µL of inactivated monomer solution with 10µL of polymerization initiators.

Embryo-embedded gels were transferred to 35mm-dishes filled with denaturation buffer (200mM SDS, 50mM Tris, 200mM NaCl in ddH20; pH9) and incubated 10 min at RT at 100rpm, prior to being transferred to individual 1.5mL tubes filled with denaturation buffer and incubated 72 hrs at 70°C. After denaturation, gels were equilibrated in an excess of denaturation-like buffer (50mM Tris, 200mM NaCl in ddH20; pH9) for 15 min at RT at 100rpm, then for 15min in PBS.

To control embedding efficiency and specimen integrity, gels were labelled for 2hrs in PBS-DAPI (1:1000), then washed 2 × 15min in PBS and observed using a Leica epifluorescence microscope. After initial validation, gels were stained in an excess of PBS-BSA 2% with primary antibodies (1:100) for 96 hrs at 37°C at 100rpm. Gels were washed 3x 1hr in PBS-Tween 0.1% (PBS-T) at RT at 100rpm and incubated in secondary antibodies (1:250) for 72 hrs at 37°C and 100rpm. After staining, gels were washed 3x 1hr in PBS-T at RT at 100rpm and equilibrated 15 min at RT in PBS. The diameter of each PBS-equilibrated gel was measured (in average 24–28mm) and a second control of labelling efficiency was performed via epifluorescence microscopy, allowing to visualize the position of the embryo and to trim gel excess. Stained gels were stable in PBS and covered from light at 4°C for up to a week and were expanded the day prior imaging.

For expansion, gels were immersed at RT in an excess of ddH20 for 2 × 30min then overnight. Expanded gels were measured the next day to define the gel expansion factor (Gel ExF). Note that gel ExF variability for TissUExM is similar to previous expansion of tissue sections or whole organisms (Mercey et al., 2022; Yu et al., 2020), but higher than previous U-ExM studies on isolated organelles or cells.

Individual gels were mounted in an Attofluor cell chamber (Thermofisher) and image acquisition was performed on a LEICA SP8 microscope. To image the whole embryo, a 10X/0.40 dry objective was used, with confocal acquisition overlapping multiple tiles (30 tiles in average), a pixel size of 2.27µm and a step-size ranging from 20µm to 50µm, using a "smooth" merge for image reconstruction. Alternatively, we used a 20X/0.75 dry objective to image smaller selected regions. For ultrastructural analysis, a 63X/1.20 water objective was used, with a step size of 0.40µm. For optional deconvolution, the LEICA "Lightening" mode was used, with "adaptive" strategy favouring best resolution, pinhole opening between 0.5 and 0.75, average from 2 to 4, pixel size of 35nm and step-size of 0.40µm. Please note that the working distance of the 63X/1.20 objective is a limiting factor and that the same gel can be imaged from different orientations to maximize access to various tissues.

Image reconstruction and analysis were performed using Fiji (Schindelin et al., 2012).

Collagenase VII digestion

For ZF older than 2dpf, an additional collagenase VII digestion was integrated to the TissUExM process, similarly to Yu et al. (2020). At the end of gelation, individual gels were transferred to 1.5mL Epi-tubes containing 1000U of Collagenase VII diluted in 1mL calcium-containing buffer (50mM Tris, 200mM NaCl, 40mM $CaCl_2$) and incubated overnight at 37C. Note that the presence of calcium prevents from gel expansion and mechanical tensions prior to specimen digestion and denaturation. After digestion, gels were transferred to 35mm dishes and washed 2 × 15min in an excess of calcium-containing buffer at 100 rpm at RT. Gels were then washed 10min in an excess of PBS and processed for denaturation.

TissUExM of *D. melanogaster* wings

Each step of TissUExM until gelation was performed on coverslips. Fixed individual wings were positioned on PDL-coated 12mm-coverslips under a binocular microscope to control the wing polarity during mounting. Crosslinking was performed by immersing the coverslip in 1mL crosslinking solution and incubation time could be reduced to 5hrs. Coverslips were carefully washed under a

binocular microscope to avoid wing detachment and incubated in inactivated gelation solution overnight similarly to ZF embryos. Coverslips were washed again under a binocular microscope prior to gelation, where coverslips were flipped on top of a drop of 50μL gelation solution. Each following step was performed similarly to ZF gels, except that the denaturation time was reduced to 20hrs.

QUANTIFICATION AND STATISTICAL ANALYSIS

Isotropy validation at the macroscale in ZF embryo

Rehydrated Tg(b-actin:Arl13B-GFP) 2dpf embryos were stained with DAPI (1:1000) in PBS-Triton 0.1% overnight at RT with orbital shaking and washed 3 × 15min in PBS-Triton0.1%. Individual embryos were mounted in a drop of PBS in a Attofluor cell chamber and confocal-tiling acquisition was performed with a step-size of 5μm and a pixel size of 2.27μm to obtain pre-TissUExM images.

Embryos were then individually processed by TissUExM, including post-expansion anti-GFP immunostaining and a second labelling step with DAPI. Confocal tiling acquisition was performed with a step-size of 10μm and a pixel size of 2.27μm.

Distortion analysis

Macroscopic distortion analysis was performed using the method described by Truckenbrodt et al., (2019). In brief, we manually generated pairs by selecting pre-TissUExM images containing the fields of view imaged post-TissUExM within regions containing clearly identified landmarks such as the excretory canal or the tail of the embryo. For the analysis, we used the 2D sum projection of the confocal 3D volumes obtained from the DAPI channel, using the stack projection tool in Fiji. Both pre-TissUExM and post-TissUExM images were made of 512 × 512 pixels representing ~1165 × 1165 μm^2 and ~291 × 291 μm^2, respectively.

Using the code provided by Truckenbrodt et al., (2019), we first found the position of the post-TissUExM image within the pre-TissUExM image, in-plane rotation angle and expansion factor. This step uses the correlation-based template matching feature of the Python package scikit-image (https://scikit-image.org/) to find the position of a pre-rotated and scaled post-TissUExM image. For each pair, a range of expansion factors from 3.8 to 4.4 in steps of 0.1 and a range of in-plane rotation angle within +/− 10 degrees of the expected angle (roughly assessed visually) in steps of 0.5 degrees were typically explored to find the match with the best correlation factor. To account for the difference in resolution between the two images, a gaussian smoothing of the post-TissUExM image was applied using a standard deviation of 1 pixel. Once the matching pair was found, the distortion vectors were calculated with using the Gunnar Farneback's dense optical flow algorithm from the Python package OpenCV (https://docs.opencv.org/) at the resolution of the post-TissUExM image. As was done in the initial method, we applied a gaussian smoothing to the post-TissUExM image using a standard deviation of 4 pixels prior to the calculation of the distortion vectors, corresponding to the expansion factor.

From the distortion vectors amplitude obtained using the Truckenbrodt et al., (2019) method, we additionally calculated the root-mean-square distortion amplitude (here referred to as root-mean-square error, or RMSE) as a function of the distance from the centre of the image pair, as is commonly done in the ExM field. For each matching pair, the RSME plot was generated over a 100μm (pre-expansion) distance. Average and standard deviation of individual RSME over 100μm was plotted on GraphPad Prism.

All code is available from the supplemental information of the Truckenbrodt et al., (2019). We noticed that the requirements.txt file was missing from the supplemental information, so we created one including the following packages: tqdm, scipy, scikit-image, jupyter and matplotlib.

Isotropy validation at the nanoscale in ZF and mouse embryos

Basal bodies were used as intracellular molecular rulers, with a minimum of 5 basal bodies/tissue/embryo. Quantification of individual basal body sizes was performed similarly to Steib et al. (2020). In brief, length and width were measured on Z-projections of basal-bodies imaged in side-view. A PolyE fluorescence intensity profile was generated using Fiji (line scan:50) along the axis of interest (proximo-distal until the transition zone where PolyE is excluded for length, transverse for width). The two most external peaks were identified to define x1 and x2 (in μm), the respective positions corresponding to 50% intensity of the external peaks. Individual lengths and widths in nm were generated using the formula (x2-x1)/gelExF*1000.

For centrioles roundness and diameter, only specimens imaged in perfect top views were used, pooling centrioles stained with AcTub and DM1a. Roundness was measured using the Fiji roundness tool, where a polygon was manually drawn by connecting the centres of fluorescence intensity along the centriole perimeter, generating a value between 0 and 1. For diameter, each centriole was analysed with two perpendicular plot profiles (line scan:10),where (x1 and x2) and (x3 and x4) respectively corresponds to the position of the maximum Y value in each fluorescence intensity peak. Individual diameters in nm were calculated using the formula [(x2-x1)+(x4-x3)]/2/gelExF*1000.

Measurements of ciliary features in ZF and mouse

Ciliary measurements were performed similarly to basal body measurements, imaged in side-view. For ciliary length, a plot profile (line scan:50) was generated with a proximo-distal axis, from the transition zone where PolyE is excluded to the tip of the cilium. Cilium width was measured with a transverse plot profile (line scan:50) at the tip of the cilium.

Measurement in *D. melanogaster* wings

As wing cells are not ciliated, we used cycling centrioles for nanoscale measures. We imaged the apical side of the pouch, the site of cell divisions, where centrioles are enriched in top view orientation. Measurement of centriole roundness and tubulin diameter was performed similarly to ZF centrioles.

Measurements of fluorescence intensity for staining in mouse embryos

For each organelle, a representative inset was chosen. A plot profile was generated via Fiji (line scan:10) over a 2μm distance, corresponding to a pre-ExM distance of 450nm.

Statistics and reproducibility

No statistical method was used to estimate sample size. The comparison of two groups was performed using a two-sided Welch t-test when normality was granted by Kolmogorov-Smirnov, or its non-parametric correspondent Mann-Whitney test. The comparison of more than two groups was performed using one-way ANOVA Kruskal-Wallis tests. N corresponds to independent biological replicates from various tissues. Every experiment was performed at least three times independently on different biological samples. Data are represented as scatter dot plot with centre line as mean with errors bars indicating standard deviations (SD). The significance level is denoted as usual *p<0.05, **p<0.01, ***p<0.001, ****p<0.0001, with exact p-values found in respective legends. All statistical analysis were performed using GraphPadPrism. Schematic representations of ZF embryos were generated via Biorender with publication licence UH238F0K80.

Cell Reports Methods, Volume *2*

Supplemental information

TissUExM enables quantitative ultrastructural analysis in whole vertebrate embryos by expansion microscopy

Emmanuelle Steib, Rob Tetley, Romain F. Laine, Dominic P. Norris, Yanlan Mao, and Julien Vermot

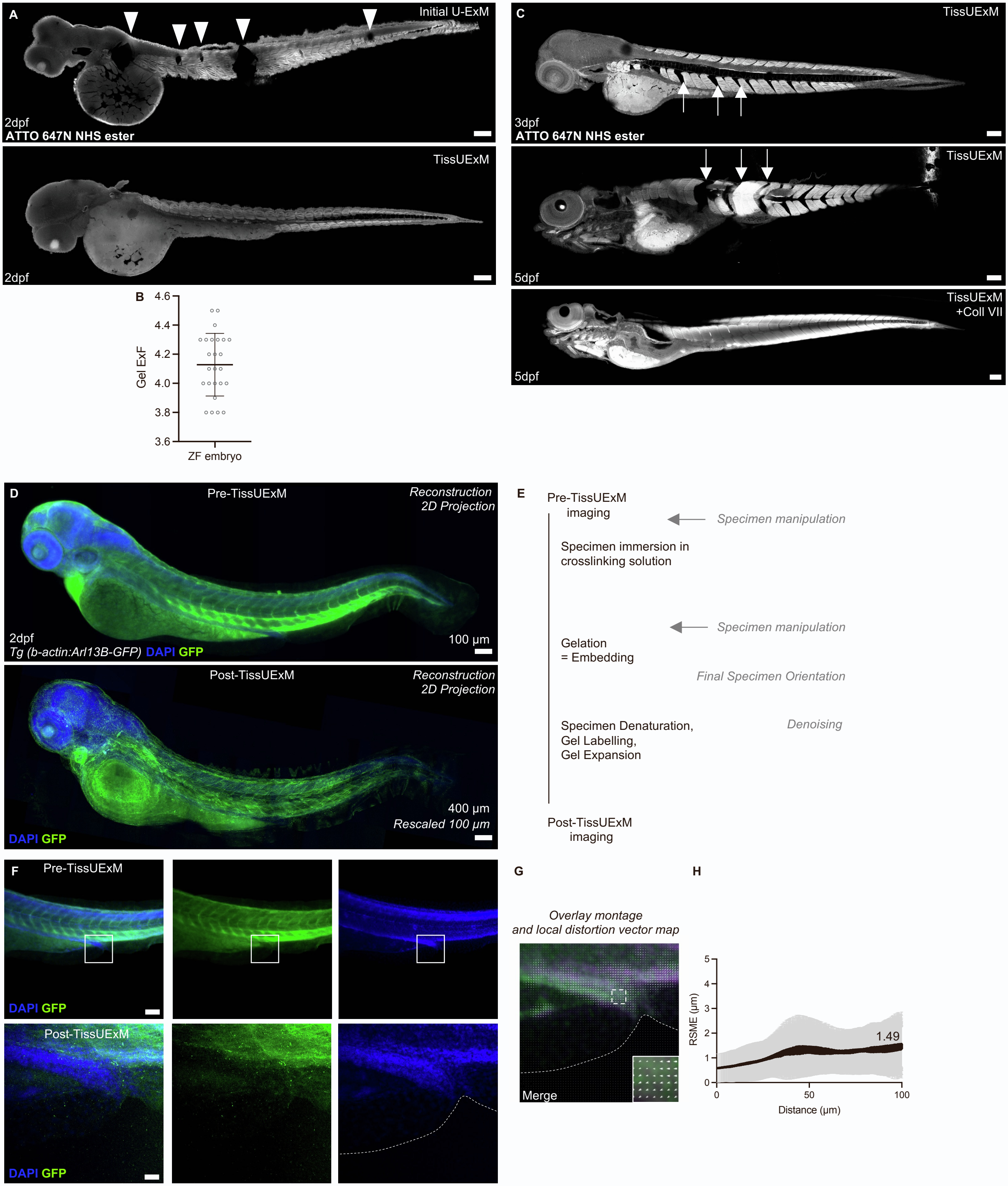

Supplemental Figure 1

Figure S1: TissUExM allows expansion of whole ZF embryos, Related to Figure 1

(A) 48hpf ZF processed with the initial U-ExM approach (top panel) or TissUExM (bottom panel), stained with ATTO 647N NHS-ester. White arrows point to cracking and specimen damage. Note that cracks are randomly localized. 10X/0.40 objective, scale bar: 100µm.
(B) Reproducibility of gel expansion factors for TissUExM processed 48hpf ZF embryo. Mean +/- SD: 4.1 +/-0.2, n=25 from 11 independent experiments.
(C) 3 to 5dpf ZF processed with basic TissUExM (top panels) or TissUExM+Collagenase VII digestion (bottom panel), stained with ATTO 647N NHS-ester. White arrows point to cracking and specimen damage. Note that cracks are restricted to myotendinous regions. 10X/0.40 objective, scale bar: 100µm, n=12 with 2 specimen/developmental stage/experiment, from 2 independent experiments.
(D) 48hpf Tg (b-actin:Arl13B-GFP) ZF imaged pre- (top panel) and post- (bottom panel) TissUExM, stained with DAPI. Entire specimen reconstruction and 2D-projection obtained from confocal imaging performed on individual tiles. 10X/0.40 objective. Pre-TissUExM corresponds to direct embryo imaging while post-TissUExM is obtained from gel-imaging. Note the appearance of the yolk-sac pre-TissUExM, with heterogenous protein density resulting from PFA-fixation.
(E) Pipeline recapitulating embryo manipulation during TissUExM.
(F) Example of the excretory canal as landmark pair for pre-(top) and post (bottom)-TissUExM distortion analysis. Inset in pre-TissUExM image corresponds to the expanded region displayed in the post-TissUExM image. 10X/0.40 objective, scale bars are respectively 100µm and 25µm.
(G) Overlay montage based on DAPI labelling, with post-TissUExM image automatically identified within pre-TissUExM image. Inset shows individual vectors for distortion analysis.
(H) Distortion analysis with individual Root Square Method Error (RSME, µm) for rescaled distance (µm). Mean+/- SD value 100µm away from centre: 1.49 +/- 0.9µm, n=20 pairs from two independent experiments. Note that RSME value at centre differs from 0 due to tilt in 3D specimen orientation between pre- and post-imaging.

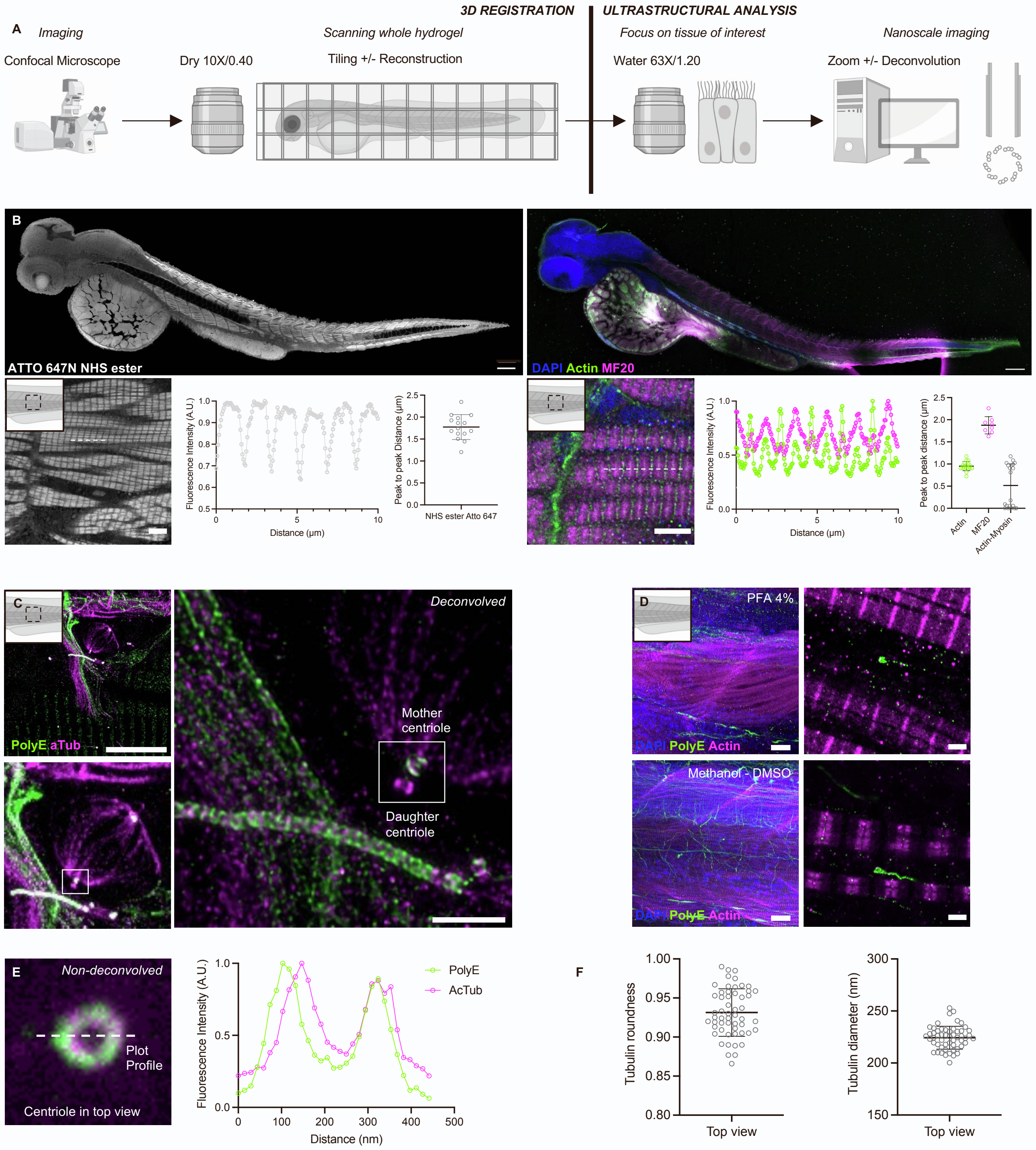

Supplemental Figure 2

Figure S2: TissUExM allows immunolabelling of whole ZF embryos and imaging at the nanoscale, Related to Figure 2

(A) Schematic representation of confocal imaging pipeline with respective objectives for different magnifications and degrees of details. Note that 63X working distance is limiting so multiple gel orientations should be considered. One gel can be imaged from different sides to maximize access to tissues of interest.
(B) TissUExM processed 48hpf ZF, labelled with ATTO 647N NHS ester (left panel) and immuno-stained for actin (green) and sarcomere myosin heavy chain (MF20, magenta) (right panel). 10X/0.40 objective, scale bar: 100µm. Inset on the sarcomeres and measurement of fluorescence intensity. Scale bar: 5µm. Mean +/- SD peak-to-peak distance with NHS labelling: 1.8 +/- 0.3 µm, n=15 distances from measures in three different sarcomeres. Mean +/- SD peak-to-peak distance with acto-myosin labelling: respectively between actin 0.9 +/-0.1µm, MF20 1.8 +/- 0.2µm and between acto-myosin.
(C) Trunk from TissUExM processed 48hpf ZF, stained for PolyE (green) and alpha-tubulin (magenta). Focus on mitotic cell near the spinal cord. Inset on deconvoluted image shows individual centrioles within the centrosome, with PolyE-positive mother centriole and PolyE-negative daughter centriole. 63X/1.20 objective, scale bars: 10µm, 1µm.
(D) Trunk from TissUExM-processed ZF embryos (10X), fixed at 48hpf either with PFA 4% (top panel) or Methanol 80%-DMSO 20% (bottom panel). Both specimen-embedded gels are processed in parallel and stained with PolyE (green) or Actin (magenta). Insets on sarcomeres and primary cilia (63X), showing that epitope conservation for individual antibodies is differentially affected by pre-TissUExM fixation, independently of gel processing. Scale bars: 20µm, 1µm.
(E) Representative TissUExM centriole in top view, with lumen resolved without deconvolution and associated plot profile.
(F) Validation of nanoscale isotropy. Mean +/- SD centriole roundness: 0.9313 +/- 0.03. n=54 centrioles from five independent experiments. Mean +/- SD centriole diameter: 224 +/- 11nm, n=54 centrioles from five independent experiments.

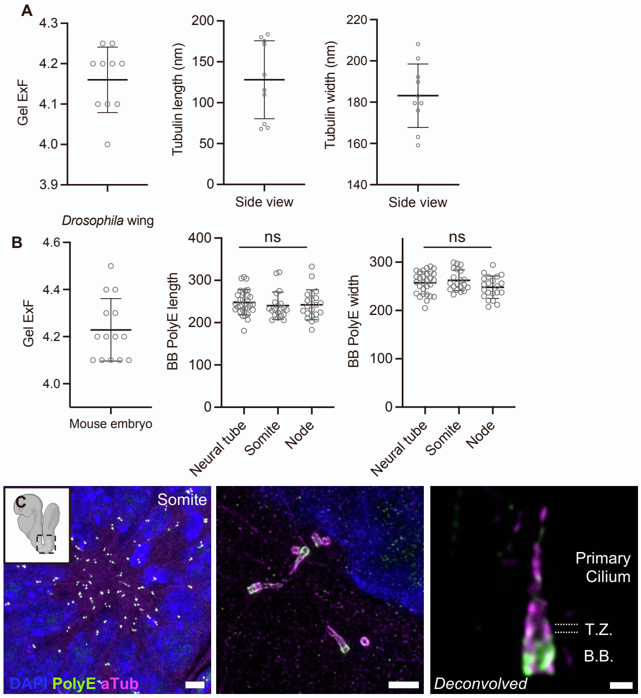

Supplemental Figure 3

Figure S3: TissUExM permits nanoscale expansion for different developmental models, Related to Figure 3

(A) Expansion validation in *Drosophila* wing discs. Reproducible expansion with Gel ExF mean +/- SD: 4.16+/- 0.1. n=10 gels from three independent experiments. Mean +/- SD centriolar tubulin length in side-view: 128+/- 48nm, n=10 centrioles from three independent experiments. Mean +/- SD centriolar tubulin width in side-view: 183 +/- 15nm, n=10 centrioles from three independent experiments.
(B) Expansion validation in mouse embryos. Reproducible expansion with Gel ExF mean +/- SD: 4.22 +/- 0.1. n=14 gels from six independent experiments. Mean +/- SD Basal body PolyE length respectively in neural tube 248 +/- 30nm, somite 240 +/- 32nm and node 242 +/- 35nm, n≥20 centriole/tissue from three independent experiments One-way ANOVA and Kruskal- Wallis ns p= 0.3532. Mean +/- SD Basal body PolyE width respectively in neural tube 257 +/- 23nm, somite 262 +/- 21nm and node 248 +/- 23nm, n≥20 centriole/tissue from three independent experiments One-way ANOVA ns p= 0.1235.
(C) Confocal imaging of a E8.5 mouse somite co-stained for PolyE (green) and a-tub (magenta). DAPI is in blue. From left to right: centre of the somite enriched in basal bodies (10X), multiple basal body/cilia complexes and inset on a primary cilium (63X). Scale bars respectively: 5µm, 1µm and 200nm.

Supplemental Tables

**Protocol Name**	**Models**	**Whole organism**	**Isotropy mm-µm-nm**	**Commercial antibodies**	**High density labelling of endogenous proteins**	**Ultra-structure**	**Standard protocol across applications**
Accepted publications							
ExM, Chen 2015	100µm mice brain sections, cultured cells	-	YES	-	-	YES	-
MAP, Ku 2016	Dissected organs from 8-weeks mice, cultured cells	-	Limited to mm-µm	YES	YES	-	-
ProExM, Tillberg 2016	100µm mice brain sections, 10µm human tissues sections, cultured cells,	-	YES	YES	-	YES	-
ExM zebrafish, Freifeld 2017	Dissected zebrafish heads	-	Limited to mm-µm	Limited	-	-	-
U-ExM, Gambarotto 2019	Isolated organelles, protists, cultured cells	-	Limited to µm-nm	YES	YES	YES	-
TRex, Damstra 2021	100µm mice brain sections, cultured cells	-	Limited to µm-nm	YES	YES	YES	-
Cryo-ExM, Laporte 2022	Cultured cells	-	Limited to µm-nm	YES	YES	YES	YES
Modified U-ExM, Mercey 2022	Mice retinal sections	-	Limited to µm-nm	YES	YES	YES	-
Preprints							
Whole ExM, Sim 2021, updated 2022	100µm mice brain sections, whole zebrafish embryos, whole mouse embryo	YES	Limited to mm-µm	Limited	-	-	-
TissUExM, This study	Whole zebrafish embryos, *Drosophila* wings, whole mouse embryo	YES	YES	YES	YES	YES	YES

Table S1: Summary description of major ExM protocols relevant for this study and rational for the development of TissUExM, Related to Figure 1

	Crosslinking	Embedding	Denaturation	Damage	Damage observation
U-ExM	1.4%FA, 2%AA - 5hrs	0.5% TEMED, 0.5% APS 5min + 1hr	90min - 95C	Major, irrespective of regions	Uncleared specimen, between gelation and denaturation, amplified by expansion
V2	↑AA concentration (20%)	↓ TEMED and APS ↑ Time	↓ Temperature ↑ Time (20hrs)	Important, mainly in the trunk	Uncleared specimen, between gelation and denaturation
V3	-	↑ 4-OH-TEMPO	-	Important, mainly in the trunk	Uncleared specimen, between gelation and denaturation
V4	↑Triton (0.1%)	↑Triton (0.1%)	-	Variable, restricted to deep trunk tissues	Cleared specimen, after expansion and NHS-labelling
V5	-	-	↑ Time (72hrs)	Variable, mostly in the centre of the tail	Cleared specimen, after expansion and NHS-labelling
V6	↑ Time (24hrs)	-	-	Minimal, restricted to the tail	Cleared specimen, after expansion and NHS-labelling
TissUExM	↑ Time (72hrs)	-	-	No damage	N/A

Table S2: Technical iterations to develop TissUExM , Related to STAR methods
